# Supplementary material for: Population Diversity and Genetic Structure Reveal Patterns of Host Association and Anthropogenic Impact for the Globally Important Fungal Tree Pathogen Ceratocystis manginecans
Source: J Fungi (Basel). 2021 Sep 15;7(9):759. doi: 10.3390/jof7090759 (PMC8470894; doi:10.3390/jof7090759)
Supplement: Supplementary file 1 [file jof-07-00759-s001.zip › jof-1374724-supplementary.pdf]

Supplementary Materials:

Supplementary Figure S1. Linkage disequilibrium testing (I_A, P-values > 0.01).

Supplementary Table S1. List of *Ceratocystis* isolates included in the microsatellite study and the alleles scored at each locus using GeneScan™ fragment analyses and the GeneMapper™ software.

Supplementary Table S2. List of *Ceratocystis* isolates included in the microsatellite study and the alleles scored at each locus based on results obtained by sequencing.

Supplementary Table S3. GenBank numbers of alleles sequenced in this study.

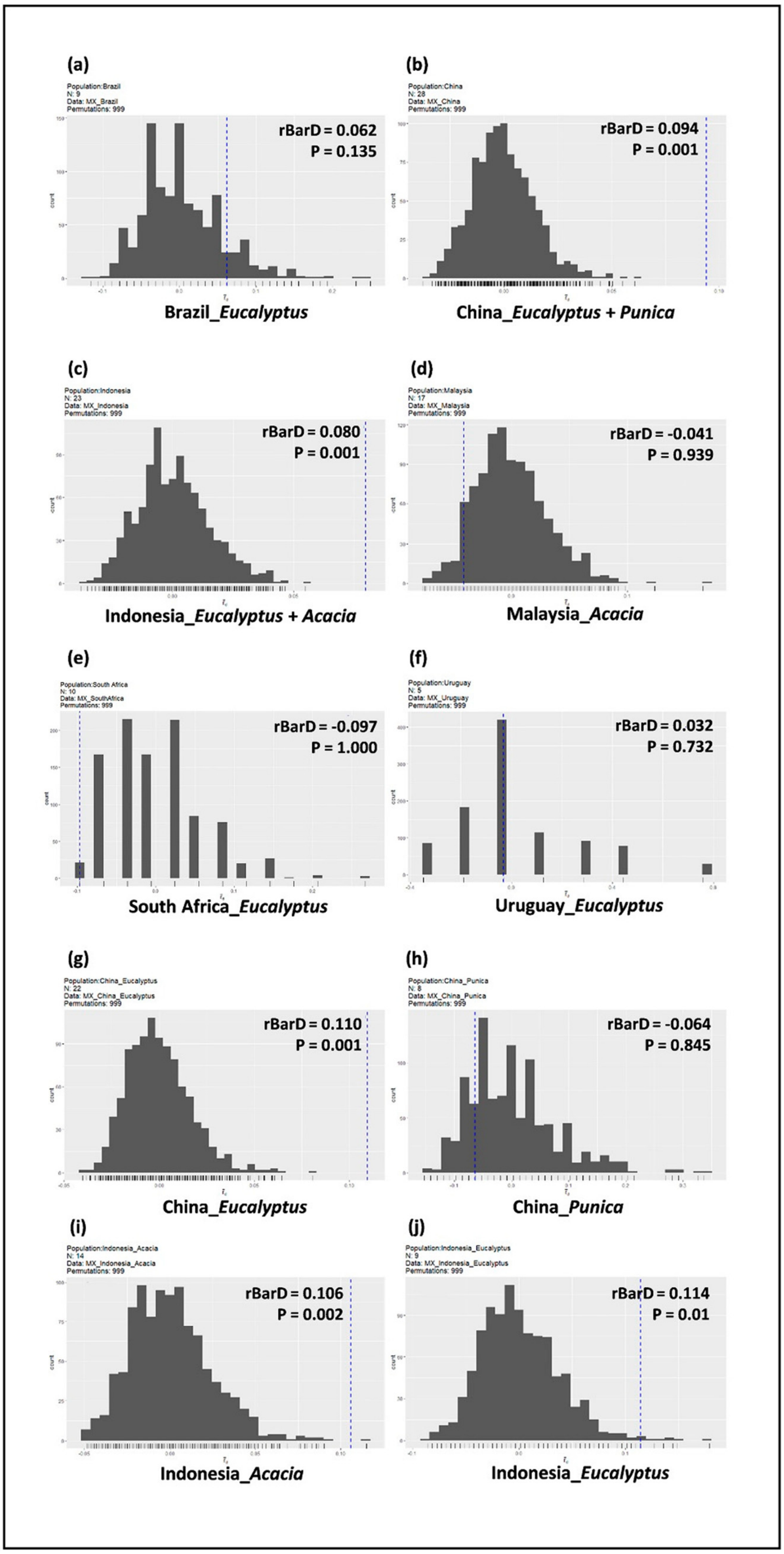

Supplementary Figure S1.

Supplementary Table S1. List of *Ceratocystis* isolates included in the microsatellite study and the alleles scored at each locus using GeneScan analyses.

					Panel AF									
				**Primer**	**AF2**	**AF3**	**AF4**	**AF5**	**AF6**	**AF7**	**AF8**	**AF9**	**AF11**	**AF12**
				**Dye**	**VIC**	**NED**	**PET**	**FAM**	**VIC**	**NED**	**PET**	**FAM**	**VIC**	**PET**
				**Colour**	**Green**	**Yellow**	**Red**	**Blue**	**Green**	**Yellow**	**Red**	**Blue**	**Green**	**Red**
				**Bin size in Genescan**	**180~230**	**170~270**	**200~280**	**200~300**	**235~320**	**280~370**	**310-380**	**350~460**	**420~490**	**400-480**
				**Repeat type**	**(AGA)n**	**(CTG)n**	**(ACA)n**	**(GTCA)n**	**(GAG)n**	**(AGC)n**	**(GAG)n**	**(GCA)n**	**(ACA)n**	**(ACA)n**
				**Dilution**	**1/200**	**1/200**	**1.2/200**	**2.5/200**	**1/200**	**1.2/200**	**1/200**	**1/200**	**1/200**	**1/200**
				**Annealing temperature**	**55°C**	**55°C**	**55°C**	**54°C**	**54°C**	**55°C**	**54°C**	**54°C**	**55°C**	**57°C**
				**Taq**	**My Taq**	**My Taq**	**My Taq**	**My Taq**	**My Taq**	**My Taq**	**My Taq**	**My Taq**	**My Taq**	**My Taq**
Nr	**Isolate[a]**	**Species**	**Location**	**Host**										
1	CMW22563	*C. manginecans*	Indonesia	*Acacia mangium*	201	218	249	262	300	325	349	412	455	453
2	CMW22561	*C. manginecans*	Indonesia	*A. mangium*	201	218	249	262	300	322	349	412	455	453
3	CMT66	*C. manginecans*	Indonesia	*A. acacinofora*	206	218	249	262	309	325	349	412	447	453
4	CMW22564	*C. manginecans*	Indonesia	*A. mangium*	206	218	243	262	300	325	349	412	447	453
5	CMT25	*C. manginecans*	Indonesia	*A. acacinofora*	206	218	243	262	300	325	349	412	447	459
6	CMT26	*C. manginecans*	Indonesia	*A. acacinofora*	206	218	243	262	300	325	349	412	447	459
7	CMT29	*C. manginecans*	Indonesia	*A. acacinofora*	206	218	243	262	300	325	349	412	447	459
8	CMT1	*C. manginecans*	Indonesia	*A. acacinofora*	206	218	243	262	300	325	349	418	447	453
9	CMT10	*C. manginecans*	Indonesia	*A. acacinofora*	206	218	243	262	300	325	349	418	447	453
10	CMT100	*C. manginecans*	Indonesia	*A. acacinofora*	206	218	243	262	300	325	349	418	447	453
11	CMT101	*C. manginecans*	Indonesia	*A. acacinofora*	206	218	243	262	300	325	349	418	447	453
12	CMT102	*C. manginecans*	Indonesia	*A. acacinofora*	206	218	243	262	300	325	349	418	447	453
13	CMT103	*C. manginecans*	Indonesia	*A. acacinofora*	206	218	243	262	300	325	349	418	447	453
14	CMT11	*C. manginecans*	Indonesia	*A. acacinofora*	206	218	243	262	300	325	349	418	447	453
15	CMT12	*C. manginecans*	Indonesia	*A. acacinofora*	206	218	243	262	300	325	349	418	447	453
16	CMT13	*C. manginecans*	Indonesia	*A. acacinofora*	206	218	243	262	300	325	349	418	447	453
17	CMT15	*C. manginecans*	Indonesia	*A. acacinofora*	206	218	243	262	300	325	349	418	447	453
18	CMT16	*C. manginecans*	Indonesia	*A. acacinofora*	206	218	243	262	300	325	349	418	447	453
19	CMT17	*C. manginecans*	Indonesia	*A. acacinofora*	206	218	243	262	300	325	349	418	447	453

20	CMT18	*C. manginecans*	Indonesia	*A. acacinofora*	206	218	243	262	300	325	349	418	447	453
21	CMT2	*C. manginecans*	Indonesia	*A. acacinofora*	206	218	243	262	300	325	349	418	447	453
22	CMT20	*C. manginecans*	Indonesia	*A. acacinofora*	206	218	243	262	300	325	349	418	447	453
23	CMT21	*C. manginecans*	Indonesia	*A. acacinofora*	206	218	243	262	300	325	349	418	447	453
24	CMT22	*C. manginecans*	Indonesia	*A. acacinofora*	206	218	243	262	300	325	349	418	447	453
25	CMT24	*C. manginecans*	Indonesia	*A. acacinofora*	206	218	243	262	300	325	349	418	447	453
26	CMT28	*C. manginecans*	Indonesia	*A. acacinofora*	206	218	243	262	300	325	349	418	447	453
27	CMT3	*C. manginecans*	Indonesia	*A. acacinofora*	206	218	243	262	300	325	349	418	447	453
28	CMT30	*C. manginecans*	Indonesia	*A. acacinofora*	206	218	243	262	300	325	349	418	447	453
29	CMT31	*C. manginecans*	Indonesia	*A. acacinofora*	206	218	243	262	300	325	349	418	447	453
30	CMT33	*C. manginecans*	Indonesia	*A. acacinofora*	206	218	243	262	300	325	349	418	447	453
31	CMT35	*C. manginecans*	Indonesia	*A. acacinofora*	206	218	243	262	300	325	349	418	447	453
32	CMT36	*C. manginecans*	Indonesia	*A. acacinofora*	206	218	243	262	300	325	349	418	447	453
33	CMT37	*C. manginecans*	Indonesia	*A. acacinofora*	206	218	243	262	300	325	349	418	447	453
34	CMT38	*C. manginecans*	Indonesia	*A. acacinofora*	206	218	243	262	300	325	349	418	447	453
35	CMT39	*C. manginecans*	Indonesia	*A. acacinofora*	206	218	243	262	300	325	349	418	447	453
36	CMT5	*C. manginecans*	Indonesia	*A. acacinofora*	206	218	243	262	300	325	349	418	447	453
37	CMT6	*C. manginecans*	Indonesia	*A. acacinofora*	206	218	243	262	300	325	349	418	447	453
38	CMT7	*C. manginecans*	Indonesia	*A. acacinofora*	206	218	243	262	300	325	349	418	447	453
39	CMT8	*C. manginecans*	Indonesia	*A. acacinofora*	206	218	243	262	300	325	349	418	447	453
40	CMT9	*C. manginecans*	Indonesia	*A. acacinofora*	206	218	243	262	300	325	349	418	447	453
41	CMT94	*C. manginecans*	Indonesia	*A. acacinofora*	206	218	243	262	300	325	349	418	447	453
42	CMT95	*C. manginecans*	Indonesia	*A. acacinofora*	206	218	243	262	300	325	349	418	447	453
43	CMT96	*C. manginecans*	Indonesia	*A. acacinofora*	206	218	243	262	300	325	349	418	447	453
44	CMT98	*C. manginecans*	Indonesia	*A. acacinofora*	206	218	243	262	300	325	349	418	447	453
45	CMT99	*C. manginecans*	Indonesia	*A. acacinofora*	206	218	243	262	300	325	349	418	447	453
46	CMT14	*C. manginecans*	Indonesia	*A. acacinofora*	206	218	243	262	300	325	349	418	447	999
47	CMT4	*C. manginecans*	Indonesia	*A. acacinofora*	206	218	243	262	300	325	349	418	447	999
48	CMW22587	*C. manginecans*	Indonesia	*A. mangium*	206	218	243	262	294	325	349	418	447	453
49	CMW22588	*C. manginecans*	Indonesia	*A. mangium*	206	218	243	262	294	325	349	418	447	453
50	CMW22589	*C. manginecans*	Indonesia	*A. mangium*	206	218	243	262	294	325	349	418	447	453
51	CMW22590	*C. manginecans*	Indonesia	*A. mangium*	206	218	243	262	294	325	349	418	447	453
52	CMW22591	*C. manginecans*	Indonesia	*A. mangium*	206	218	243	262	294	325	349	418	447	453

53	CMW22593	*C. manginecans*	Indonesia	*A. mangium*	206	218	243	262	294	325	349	418	447	453
54	CMW22594	*C. manginecans*	Indonesia	*A. mangium*	206	218	243	262	294	325	349	418	447	453
55	CMW22595	*C. manginecans*	Indonesia	*A. mangium*	206	218	243	262	294	325	349	418	447	453
56	CMW22596	*C. manginecans*	Indonesia	*A. mangium*	206	218	243	262	294	325	349	418	447	453
57	CMW22597	*C. manginecans*	Indonesia	*A. mangium*	206	218	243	262	294	325	349	418	447	453
58	CMW22598	*C. manginecans*	Indonesia	*A. mangium*	206	218	243	262	294	325	349	418	447	453
59	CMW22618	*C. manginecans*	Indonesia	*A. mangium*	206	218	243	262	294	325	349	418	447	456
60	CMW22619	*C. manginecans*	Indonesia	*A. mangium*	206	218	243	262	294	325	349	418	447	456
61	CMW22621	*C. manginecans*	Indonesia	*A. mangium*	206	218	243	262	294	325	349	418	447	456
62	CMW22622	*C. manginecans*	Indonesia	*A. mangium*	206	218	243	262	294	325	349	418	447	456
63	CMW22623	*C. manginecans*	Indonesia	*A. mangium*	206	218	243	262	294	325	349	418	447	456
64	CMW22625	*C. manginecans*	Indonesia	*A. mangium*	206	218	243	262	294	325	349	418	447	456
65	CMW22626	*C. manginecans*	Indonesia	*A. mangium*	206	218	243	262	294	325	349	418	447	456
66	CMT64	*C. manginecans*	Indonesia	*A. acacinofora*	206	218	243	262	294	325	349	418	447	456
67	CMT65	*C. manginecans*	Indonesia	*A. acacinofora*	206	218	243	262	294	325	349	418	447	456
68	CMT67	*C. manginecans*	Indonesia	*A. acacinofora*	206	218	243	262	294	325	349	418	447	456
69	CMT32	*C. manginecans*	Indonesia	*A. acacinofora*	206	218	243	262	294	325	346	418	447	453
70	CMW22581	*C. manginecans*	Indonesia	*A. mangium*	206	221	243	262	294	325	349	418	447	453
71	CMW22560	*C. manginecans*	Indonesia	*A. mangium*	206	221	243	262	294	325	346	418	447	456
72	CMW22565	*C. manginecans*	Indonesia	*A. mangium*	206	221	243	262	294	325	346	418	447	456
73	CMW22566	*C. manginecans*	Indonesia	*A. mangium*	206	221	243	262	294	325	346	418	447	456
74	CMW22567	*C. manginecans*	Indonesia	*A. mangium*	206	221	243	262	294	325	346	418	447	456
75	CMW22568	*C. manginecans*	Indonesia	*A. mangium*	206	221	243	262	294	325	346	418	447	456
76	CMW22569	*C. manginecans*	Indonesia	*A. mangium*	206	221	243	262	294	325	346	418	447	456
77	CMW22570	*C. manginecans*	Indonesia	*A. mangium*	206	221	243	262	294	325	346	418	447	456
78	CMW22571	*C. manginecans*	Indonesia	*A. mangium*	206	221	243	262	294	325	346	418	447	456
79	CMW22572	*C. manginecans*	Indonesia	*A. mangium*	206	221	243	262	294	325	346	418	447	456
80	CMW22573	*C. manginecans*	Indonesia	*A. mangium*	206	221	243	262	294	325	346	418	447	456
81	CMW22574	*C. manginecans*	Indonesia	*A. mangium*	206	221	243	262	294	325	346	418	447	456
82	CMW22575	*C. manginecans*	Indonesia	*A. mangium*	206	221	243	262	294	325	346	418	447	456
83	CMW22576	*C. manginecans*	Indonesia	*A. mangium*	206	221	243	262	294	325	346	418	447	456
84	CMW22577	*C. manginecans*	Indonesia	*A. mangium*	206	221	243	262	294	325	346	418	447	456
85	CMW22578	*C. manginecans*	Indonesia	*A. mangium*	206	221	243	262	294	325	346	418	447	456

86	CMW22579	*C. manginecans*	Indonesia	*A. mangium*	206	221	243	262	294	325	346	418	447	456
87	CMW22580	*C. manginecans*	Indonesia	*A. mangium*	206	221	243	262	294	325	346	418	447	456
88	CMW22582	*C. manginecans*	Indonesia	*A. mangium*	206	221	243	262	294	322	349	418	447	453
89	CMW22583	*C. manginecans*	Indonesia	*A. mangium*	206	221	243	262	294	322	349	418	447	453
90	CMW22584	*C. manginecans*	Indonesia	*A. mangium*	206	221	243	262	294	322	349	418	447	453
91	CMW22585	*C. manginecans*	Indonesia	*A. mangium*	206	221	243	262	294	322	349	418	447	453
92	CMW22600	*C. manginecans*	Indonesia	*A. mangium*	206	221	243	262	294	322	349	418	447	453
93	CMW22601	*C. manginecans*	Indonesia	*A. mangium*	206	221	243	262	294	322	349	418	447	453
94	CMW22603	*C. manginecans*	Indonesia	*A. mangium*	206	221	243	262	294	322	349	418	447	453
95	CMW22606	*C. manginecans*	Indonesia	*A. mangium*	206	221	243	262	294	322	349	418	447	453
96	CMW22607	*C. manginecans*	Indonesia	*A. mangium*	206	221	243	262	294	322	349	418	447	453
97	CMW22608	*C. manginecans*	Indonesia	*A. mangium*	206	221	243	262	294	322	349	418	447	453
98	CMW22609	*C. manginecans*	Indonesia	*A. mangium*	206	221	243	262	294	322	349	418	447	453
99	CMW22612	*C. manginecans*	Indonesia	*A. mangium*	206	221	243	262	294	322	349	418	447	453
100	CMW22613	*C. manginecans*	Indonesia	*A. mangium*	206	221	243	262	294	322	349	418	447	453
101	CMW22614	*C. manginecans*	Indonesia	*A. mangium*	206	221	243	262	294	322	349	418	447	453
102	CMW22615	*C. manginecans*	Indonesia	*A. mangium*	206	221	243	262	294	322	349	418	447	453
103	CMW22616	*C. manginecans*	Indonesia	*A. mangium*	206	221	243	262	294	322	349	418	447	453
104	CMW22617	*C. manginecans*	Indonesia	*A. mangium*	206	221	243	262	294	322	349	418	447	453
105	CMW22599	*C. manginecans*	Indonesia	*A. mangium*	206	221	243	250	294	322	346	418	447	456
106	CMW22602	*C. manginecans*	Indonesia	*A. mangium*	206	221	243	250	294	322	346	418	447	456
107	CMW22604	*C. manginecans*	Indonesia	*A. mangium*	206	221	243	250	294	322	346	418	447	456
108	CMW22605	*C. manginecans*	Indonesia	*A. mangium*	206	221	243	250	294	322	346	418	447	456
109	CMW22610	*C. manginecans*	Indonesia	*A. mangium*	206	221	243	250	294	322	346	418	447	456
110	CMW22611	*C. manginecans*	Indonesia	*A. mangium*	206	221	243	250	294	322	346	418	447	456
111	CMW41202	*C. manginecans*	Malaysia	*A. mangium*	201	221	243	250	294	325	349	418	447	456
112	CMW41190	*C. manginecans*	Malaysia	*A. mangium*	201	221	243	250	294	322	346	418	447	453
113	CMW42003	*C. manginecans*	Malaysia	*A. mangium*	206	218	243	250	294	322	346	418	447	453
114	CMW41203	*C. eucalypticola*	Malaysia	*A. mangium*	206	221	243	250	300	325	349	418	447	453
115	CMW39172	*C. manginecans*	Malaysia	*A. mangium*	206	221	243	250	294	325	346	412	447	453
116	CMW41147	*C. manginecans*	Malaysia	*A. mangium*	206	221	243	250	294	325	346	412	447	441
117	CMW41168	*C. manginecans*	Malaysia	*A. mangium*	206	221	243	250	294	325	346	412	447	441
118	CMW38751	*C. manginecans*	Malaysia	*A. mangium*	206	221	243	250	294	325	346	418	447	453

119	CMW38754	*C. manginecans*	Malaysia	*A. mangium*	206	221	243	250	294	325	346	418	447	453
120	CMW41155	*C. manginecans*	Malaysia	*A. mangium*	206	221	243	250	294	325	346	418	447	453
121	CMW41171	*C. manginecans*	Malaysia	*A. mangium*	206	221	243	250	294	325	346	418	447	453
122	CMW41193	*C. manginecans*	Malaysia	*A. mangium*	206	221	243	250	294	325	346	418	447	453
123	CMW41152	*C. manginecans*	Malaysia	*A. mangium*	206	221	243	250	294	325	346	418	447	441
124	CMW41160	*C. manginecans*	Malaysia	*A. mangium*	206	221	243	250	294	325	346	418	447	441
125	CMW41181	*C. manginecans*	Malaysia	*A. mangium*	206	221	243	250	294	325	346	418	447	441
126	CMW42006	*C. manginecans*	Malaysia	*A. mangium*	206	221	243	250	294	325	346	418	447	456
127	CMW38750	*C. manginecans*	Malaysia	*A. mangium*	206	221	243	250	294	322	346	418	447	453
128	CMW38753	*C. manginecans*	Malaysia	*A. mangium*	206	221	243	250	294	322	346	418	447	453
129	CMW39136	*C. manginecans*	Malaysia	*A. mangium*	206	221	243	250	294	322	346	418	447	453
130	CMW39141	*C. manginecans*	Malaysia	*A. mangium*	206	221	243	250	294	322	346	418	447	453
131	CMW39152	*C. manginecans*	Malaysia	*A. mangium*	206	221	243	250	294	322	346	418	447	453
132	CMW39155	*C. manginecans*	Malaysia	*A. mangium*	206	221	243	250	294	322	346	418	447	453
133	CMW39157	*C. manginecans*	Malaysia	*A. mangium*	206	221	243	250	294	322	346	418	447	453
134	CMW39162	*C. manginecans*	Malaysia	*A. mangium*	206	221	243	250	294	322	346	418	447	453
135	CMW39168	*C. manginecans*	Malaysia	*A. mangium*	206	221	243	250	294	322	346	418	447	453
136	CMW39171	*C. manginecans*	Malaysia	*A. mangium*	206	221	243	250	294	322	346	418	447	453
137	CMW39180	*C. manginecans*	Malaysia	*A. mangium*	206	221	243	250	294	322	346	418	447	453
138	CMW39182	*C. manginecans*	Malaysia	*A. mangium*	206	221	243	250	294	322	346	418	447	453
139	CMW41138	*C. manginecans*	Malaysia	*A. mangium*	206	221	243	250	294	322	346	418	447	453
140	CMW41149	*C. manginecans*	Malaysia	*A. mangium*	206	221	243	250	294	322	346	418	447	453
141	CMW41157	*C. manginecans*	Malaysia	*A. mangium*	206	221	243	250	294	322	346	418	447	453
142	CMW41170	*C. manginecans*	Malaysia	*A. mangium*	206	221	243	250	294	322	346	418	447	453
143	CMW41173	*C. manginecans*	Malaysia	*A. mangium*	206	221	243	250	294	322	346	418	447	453
144	CMW41174	*C. manginecans*	Malaysia	*A. mangium*	206	221	243	250	294	322	346	418	447	453
145	CMW41182	*C. manginecans*	Malaysia	*A. mangium*	206	221	243	250	294	322	346	418	447	453
146	CMW41194	*C. manginecans*	Malaysia	*A. mangium*	206	221	243	250	294	322	346	418	447	453
147	CMW39144	*C. manginecans*	Malaysia	*A. mangium*	206	221	243	250	294	322	346	418	447	441
148	CMW39149	*C. manginecans*	Malaysia	*A. mangium*	206	221	243	250	294	322	346	418	447	441
149	CMW39161	*C. manginecans*	Malaysia	*A. mangium*	206	221	243	250	294	322	346	418	447	441
150	CMW39173	*C. manginecans*	Malaysia	*A. mangium*	206	221	243	250	294	322	346	418	447	441
151	CMW39174	*C. manginecans*	Malaysia	*A. mangium*	206	221	243	250	294	322	346	418	447	441

152	CMW39176	*C. manginecans*	Malaysia	*A. mangium*	206	221	243	250	294	322	346	418	447	441
153	CMW41140	*C. manginecans*	Malaysia	*A. mangium*	206	221	243	250	294	322	346	418	447	441
154	CMW41143	*C. manginecans*	Malaysia	*A. mangium*	206	221	243	250	294	322	346	418	447	441
155	CMW41150	*C. manginecans*	Malaysia	*A. mangium*	206	221	243	250	294	322	346	418	447	441
156	CMW41164	*C. manginecans*	Malaysia	*A. mangium*	206	221	243	250	294	322	346	418	447	441
157	CMW41169	*C. manginecans*	Malaysia	*A. mangium*	206	221	243	250	294	322	346	418	447	441
158	CMW39139	*C. manginecans*	Malaysia	*A. mangium*	206	221	243	250	294	322	346	418	447	456
159	CMW41139	*C. manginecans*	Malaysia	*A. mangium*	206	221	243	250	294	322	346	418	447	459
160	CMW41148	*C. manginecans*	Malaysia	*A. mangium*	206	221	243	250	294	322	346	418	444	441
161	CMW41146	*C. manginecans*	Malaysia	*A. mangium*	206	221	243	250	294	319	346	418	447	453
162	CMW36164	*C. manginecans*	Malaysia	*A. mangium*	206	221	243	250	291	325	346	418	447	453
163	CMW39153	*C. manginecans*	Malaysia	*A. mangium*	206	221	243	250	291	325	346	418	447	453
164	CMW41142	*C. manginecans*	Malaysia	*A. mangium*	209	221	243	250	294	322	346	418	447	453
165	CMW41159	*C. manginecans*	Malaysia	*A. mangium*	209	221	243	250	294	322	346	418	447	453
166	CMW49238	*C. eucalypticola*	Brazil	*Eucalyptus* sp.	201	218	249	250	300	325	349	412	455	423
167	CMW49250	*C. eucalypticola*	Brazil	*Eucalyptus* sp.	201	218	249	250	300	325	349	415	455	444
168	CMW49237	*C. eucalypticola*	Brazil	*Eucalyptus* sp.	201	218	249	250	300	325	352	415	450	423
169	CMW49233	*C. eucalypticola*	Brazil	*Eucalyptus* sp.	201	218	249	250	300	325	352	415	450	423
170	CMW49252	*C. eucalypticola*	Brazil	*Eucalyptus* sp.	198	218	249	250	300	325	349	412	452	429
171	CMW49253	*C. eucalypticola*	Brazil	*Eucalyptus* sp.	198	218	249	250	300	325	349	412	452	429
172	CMW49239	*C. eucalypticola*	Brazil	*Eucalyptus* sp.	198	218	249	250	300	325	349	412	452	432
173	CMW49236	*C. eucalypticola*	Brazil	*Eucalyptus* sp.	198	218	249	250	300	325	349	415	452	423
174	CMW49254	*C. eucalypticola*	Brazil	*Eucalyptus* sp.	198	218	249	250	300	325	349	415	452	444
175	CMW49255	*C. eucalypticola*	Brazil	*Eucalyptus* sp.	198	218	249	250	300	325	349	415	452	444
176	CMW4903	*C. eucalypticola*	Brazil	*Eucalyptus* sp.	198	218	249	250	300	325	349	415	452	447
177	CMW49251	*C. eucalypticola*	Brazil	*Eucalyptus* sp.	198	218	249	250	300	325	349	415	452	447
178	CMW49235	*C. eucalypticola*	Brazil	*Eucalyptus* sp.	198	218	249	250	294	325	349	412	452	429
179	CERC2453	*C. eucalypticola*	China, FJ	*Eucalyptus* sp.	201	218	249	262	300	325	349	412	455	447
180	CERC2454	*C. eucalypticola*	China, FJ	*Eucalyptus* sp.	201	218	249	262	300	325	349	412	455	447
181	CERC2455	*C. eucalypticola*	China, FJ	*Eucalyptus* sp.	201	218	249	262	300	325	349	412	455	447
182	CERC2476	*C. eucalypticola*	China, FJ	*Eucalyptus* sp.	201	218	249	262	300	325	349	412	455	447
183	CERC2477	*C. eucalypticola*	China, FJ	*Eucalyptus* sp.	201	218	249	262	300	325	349	412	455	447
184	CERC2478	*C. eucalypticola*	China, FJ	*Eucalyptus* sp.	201	218	249	262	300	325	349	412	455	447

185	CERC2479	*C. eucalypticola*	China, FJ	*Eucalyptus* sp.	201	218	249	262	300	325	349	412	455	447
186	CERC2480	*C. eucalypticola*	China, FJ	*Eucalyptus* sp.	198	218	249	262	300	325	349	412	455	447
187	CERC2487	*C. eucalypticola*	China, FJ	*Eucalyptus* sp.	198	218	249	250	300	325	349	412	455	450
188	CERC2488	*C. eucalypticola*	China, FJ	*Eucalyptus* sp.	198	218	249	250	300	325	349	412	455	450
189	CERC2489	*C. eucalypticola*	China, FJ	*Eucalyptus* sp.	198	218	249	250	300	325	349	412	455	450
190	CERC2490	*C. eucalypticola*	China, FJ	*Eucalyptus* sp.	198	218	249	250	300	325	349	412	455	450
191	CERC2491	*C. eucalypticola*	China, FJ	*Eucalyptus* sp.	198	218	249	250	300	325	349	412	455	450
192	CERC2545	*C. eucalypticola*	China, GD	*Eucalyptus* sp.	201	218	249	262	300	325	343	412	455	420
193	CERC2546	*C. eucalypticola*	China, GD	*Eucalyptus* sp.	201	218	249	262	300	325	343	412	455	420
194	CERC2612	*C. eucalypticola*	China, GD	*Eucalyptus* sp.	201	218	249	262	300	325	343	412	450	447
195	CMW24667	*C. eucalypticola*	China, GD	*Eucalyptus* sp.	201	218	249	250	300	325	349	412	455	447
196	CERC2555	*C. eucalypticola*	China, GD	*Eucalyptus* sp.	201	218	249	250	300	325	349	412	450	447
197	CERC2556	*C. eucalypticola*	China, GD	*Eucalyptus* sp.	201	218	249	250	300	325	349	412	450	447
198	CERC2557	*C. eucalypticola*	China, GD	*Eucalyptus* sp.	201	218	249	250	300	325	349	412	450	447
199	CMW24664	*C. eucalypticola*	China, GD	*Eucalyptus* sp.	201	218	252	262	300	325	349	412	455	420
200	CMW24673	*C. eucalypticola*	China, GD	*Eucalyptus* sp.	201	218	252	262	300	325	349	412	455	420
201	CERC2620	*C. eucalypticola*	China, GD	*Eucalyptus* sp.	201	218	252	250	300	325	349	412	450	447
202	CERC2621	*C. eucalypticola*	China, GD	*Eucalyptus* sp.	201	218	252	250	300	325	349	412	450	447
203	CERC2629	*C. manginecans*	China, GD	*Eucalyptus* sp.	201	221	252	250	294	325	346	418	450	453
204	CERC2630	*C. manginecans*	China, GD	*Eucalyptus* sp.	201	221	252	250	294	325	346	418	450	453
205	CERC2631	*C. manginecans*	China, GD	*Eucalyptus* sp.	201	221	252	250	294	325	346	418	450	453
206	CERC2632	*C. manginecans*	China, GD	*Eucalyptus* sp.	201	221	252	250	294	325	346	418	450	453
207	CERC2633	*C. manginecans*	China, GD	*Eucalyptus* sp.	201	221	252	250	294	325	346	418	450	453
208	CERC2634	*C. manginecans*	China, GD	*Eucalyptus* sp.	201	221	252	250	294	325	346	418	450	453
209	CERC2635	*C. manginecans*	China, GD	*Eucalyptus* sp.	201	221	252	250	294	325	346	418	450	453
210	CERC2636	*C. manginecans*	China, GD	*Eucalyptus* sp.	201	221	252	250	294	325	346	418	450	453
211	CERC2637	*C. manginecans*	China, GD	*Eucalyptus* sp.	201	221	252	250	294	325	346	418	450	453
212	CERC2638	*C. manginecans*	China, GD	*Eucalyptus* sp.	201	221	252	250	294	325	346	418	450	453
213	CERC2639	*C. manginecans*	China, GD	*Eucalyptus* sp.	201	221	252	250	294	325	346	418	450	453
214	CERC2640	*C. manginecans*	China, GD	*Eucalyptus* sp.	201	221	252	250	294	325	346	418	450	453
215	CERC2641	*C. manginecans*	China, GD	*Eucalyptus* sp.	201	221	252	250	294	325	346	418	450	453
216	CERC2642	*C. manginecans*	China, GD	*Eucalyptus* sp.	201	221	252	250	294	325	346	418	450	453
217	CERC2643	*C. manginecans*	China, GD	*Eucalyptus* sp.	201	221	252	250	294	325	346	418	450	453

218	CERC2644	*C. manginecans*	China, GD	*Eucalyptus* sp.	201	221	252	250	294	325	346	418	450	453
219	CERC2645	*C. manginecans*	China, GD	*Eucalyptus* sp.	201	221	252	250	294	325	346	418	450	453
220	CERC2624	*C. manginecans*	China, GD	*Eucalyptus* sp.	206	221	249	250	300	325	346	418	447	453
221	CERC2625	*C. manginecans*	China, GD	*Eucalyptus* sp.	206	221	249	250	300	325	346	418	447	453
222	CERC2626	*C. manginecans*	China, GD	*Eucalyptus* sp.	206	221	249	250	300	325	346	418	447	453
223	CERC2589	*C. eucalypticola*	China, GD	*Eucalyptus* sp.	198	218	249	250	300	325	352	412	450	447
224	CERC2590	*C. eucalypticola*	China, GD	*Eucalyptus* sp.	198	218	249	250	300	325	352	412	450	447
225	CERC2591	*C. eucalypticola*	China, GD	*Eucalyptus* sp.	198	218	249	250	300	325	352	412	450	447
226	CERC2592	*C. eucalypticola*	China, GD	*Eucalyptus* sp.	198	218	249	250	300	325	352	412	450	447
227	CERC2613	*C. eucalypticola*	China, GD	*Eucalyptus* sp.	198	218	249	250	300	325	352	412	450	447
228	CERC2614	*C. eucalypticola*	China, GD	*Eucalyptus* sp.	198	218	249	250	300	325	352	412	450	447
229	CERC2615	*C. eucalypticola*	China, GD	*Eucalyptus* sp.	198	218	249	250	300	325	352	412	450	447
230	CERC2616	*C. eucalypticola*	China, GD	*Eucalyptus* sp.	198	218	249	250	300	325	352	412	450	447
231	CERC2617	*C. eucalypticola*	China, GD	*Eucalyptus* sp.	198	218	249	250	300	325	352	412	450	447
232	CERC2618	*C. eucalypticola*	China, GD	*Eucalyptus* sp.	198	218	249	250	300	325	352	412	450	447
233	CERC2619	*C. eucalypticola*	China, GD	*Eucalyptus* sp.	198	218	249	250	300	325	352	412	450	447
234	CERC2622	*C. eucalypticola*	China, GD	*Eucalyptus* sp.	198	218	249	250	300	325	352	412	450	447
235	CERC2623	*C. eucalypticola*	China, GD	*Eucalyptus* sp.	198	218	249	250	300	325	352	412	450	447
236	CERC2627	*C. eucalypticola*	China, GD	*Eucalyptus* sp.	198	218	252	262	300	325	349	412	455	447
237	CERC2628	*C. eucalypticola*	China, GD	*Eucalyptus* sp.	198	218	252	262	300	325	349	412	455	447
238	CERC2593	*C. eucalypticola*	China, GD	*Eucalyptus* sp.	198	218	252	262	300	325	343	412	455	447
239	CERC2594	*C. eucalypticola*	China, GD	*Eucalyptus* sp.	198	218	252	262	300	325	343	412	455	447
240	CERC2595	*C. eucalypticola*	China, GD	*Eucalyptus* sp.	198	218	252	262	300	325	343	412	455	447
241	CERC2596	*C. eucalypticola*	China, GD	*Eucalyptus* sp.	198	218	252	262	300	325	343	412	455	447
242	CERC2597	*C. eucalypticola*	China, GD	*Eucalyptus* sp.	198	218	252	262	300	325	343	412	455	447
243	CERC2598	*C. eucalypticola*	China, GD	*Eucalyptus* sp.	198	218	252	262	300	325	343	412	455	447
244	CERC2599	*C. eucalypticola*	China, GD	*Eucalyptus* sp.	198	218	252	262	300	325	343	412	455	447
245	CERC2587	*C. eucalypticola*	China, GD	*Eucalyptus* sp.	198	218	252	262	300	328	349	412	450	447
246	CERC2588	*C. eucalypticola*	China, GD	*Eucalyptus* sp.	198	218	252	262	300	328	349	412	450	447
247	CERC2606	*C. eucalypticola*	China, GD	*Eucalyptus* sp.	198	218	252	262	300	328	349	412	450	447
248	CERC2607	*C. eucalypticola*	China, GD	*Eucalyptus* sp.	198	218	252	262	300	328	349	412	450	447
249	CERC2608	*C. eucalypticola*	China, GD	*Eucalyptus* sp.	198	218	252	262	300	328	349	412	450	447
250	CERC2009	*C. eucalypticola*	China, GX	*Eucalyptus* sp.	201	218	252	250	300	325	349	412	455	450

251	CERC2010	*C. eucalypticola*	China, GX	*Eucalyptus* sp.	201	218	252	250	300	325	349	412	455	450
252	CERC2011	*C. eucalypticola*	China, GX	*Eucalyptus* sp.	201	218	252	250	300	325	349	412	455	450
253	CERC2012	*C. eucalypticola*	China, GX	*Eucalyptus* sp.	201	218	252	250	300	325	349	412	455	450
254	CERC2013	*C. eucalypticola*	China, GX	*Eucalyptus* sp.	201	218	252	250	300	325	349	412	455	450
255	CERC2016	*C. eucalypticola*	China, GX	*Eucalyptus* sp.	201	218	252	250	300	325	349	412	455	450
256	CERC2017	*C. eucalypticola*	China, GX	*Eucalyptus* sp.	201	218	252	250	300	325	349	412	455	450
257	CERC2103	*C. eucalypticola*	China, HN	*Eucalyptus* sp.	201	218	249	250	300	325	349	412	455	447
258	CERC2104	*C. eucalypticola*	China, HN	*Eucalyptus* sp.	201	218	249	250	300	325	349	412	455	447
259	CERC2105	*C. eucalypticola*	China, HN	*Eucalyptus* sp.	201	218	249	250	300	325	349	412	455	447
260	CERC2106	*C. eucalypticola*	China, HN	*Eucalyptus* sp.	201	218	249	250	300	325	349	412	455	447
261	CERC2107	*C. eucalypticola*	China, HN	*Eucalyptus* sp.	201	218	249	250	300	325	349	412	455	447
262	CERC2109	*C. eucalypticola*	China, HN	*Eucalyptus* sp.	201	218	249	250	300	325	349	412	455	447
263	CERC2110	*C. eucalypticola*	China, HN	*Eucalyptus* sp.	201	218	249	250	300	325	349	412	455	447
264	CERC2111	*C. eucalypticola*	China, HN	*Eucalyptus* sp.	201	218	249	250	300	325	349	412	455	447
265	CERC2113	*C. eucalypticola*	China, HN	*Eucalyptus* sp.	201	218	249	250	300	325	349	412	455	447
266	CERC2115	*C. eucalypticola*	China, HN	*Eucalyptus* sp.	201	218	249	250	300	325	349	412	455	447
267	CERC2068	*C. eucalypticola*	China, HN	*Eucalyptus* sp.	201	218	249	250	300	325	349	412	450	447
268	CERC2070	*C. eucalypticola*	China, HN	*Eucalyptus* sp.	201	218	249	250	300	325	349	412	450	447
269	CERC2074	*C. eucalypticola*	China, HN	*Eucalyptus* sp.	201	218	249	250	300	325	349	412	450	447
270	CERC2076	*C. eucalypticola*	China, HN	*Eucalyptus* sp.	201	218	249	250	300	325	349	412	450	447
271	CERC2079	*C. eucalypticola*	China, HN	*Eucalyptus* sp.	201	218	249	250	300	325	349	412	450	447
272	CERC2080	*C. eucalypticola*	China, HN	*Eucalyptus* sp.	201	218	249	250	300	325	349	412	450	447
273	CERC2082	*C. eucalypticola*	China, HN	*Eucalyptus* sp.	201	218	249	250	300	325	349	412	450	447
274	CERC2084	*C. eucalypticola*	China, HN	*Eucalyptus* sp.	201	218	249	250	300	325	349	412	450	447
275	CERC2085	*C. eucalypticola*	China, HN	*Eucalyptus* sp.	201	218	249	250	300	325	349	412	450	447
276	CERC2088	*C. eucalypticola*	China, HN	*Eucalyptus* sp.	201	218	249	250	300	325	349	412	450	447
277	CERC2089	*C. eucalypticola*	China, HN	*Eucalyptus* sp.	201	218	249	250	300	325	349	412	450	447
278	CERC2097	*C. eucalypticola*	China, HN	*Eucalyptus* sp.	201	218	249	250	300	325	349	412	450	447
279	CERC2101	*C. eucalypticola*	China, HN	*Eucalyptus* sp.	201	218	249	250	300	325	349	412	450	447
280	CERC2102	*C. eucalypticola*	China, HN	*Eucalyptus* sp.	201	218	249	250	300	325	349	412	450	447
281	CERC2190	*C. eucalypticola*	China, HN	*Eucalyptus* sp.	201	218	252	262	300	325	349	412	455	447
282	CERC2191	*C. eucalypticola*	China, HN	*Eucalyptus* sp.	201	218	252	262	300	325	349	412	455	447
283	CERC2192	*C. eucalypticola*	China, HN	*Eucalyptus* sp.	201	218	252	262	300	325	349	412	455	447

284	CERC2193	*C. eucalypticola*	China, HN	*Eucalyptus* sp.	201	218	252	262	300	325	349	412	455	447
285	CERC2194	*C. eucalypticola*	China, HN	*Eucalyptus* sp.	201	218	252	262	300	325	349	412	455	447
286	CERC2195	*C. eucalypticola*	China, HN	*Eucalyptus* sp.	201	218	252	262	300	325	349	412	455	447
287	CERC2196	*C. eucalypticola*	China, HN	*Eucalyptus* sp.	201	218	252	262	300	325	349	412	455	447
288	CERC2126	*C. eucalypticola*	China, HN	*Eucalyptus* sp.	201	218	252	262	300	325	343	412	455	447
289	CERC2127	*C. eucalypticola*	China, HN	*Eucalyptus* sp.	201	218	252	262	300	325	343	412	455	447
290	CERC2129	*C. eucalypticola*	China, HN	*Eucalyptus* sp.	201	218	252	262	300	325	343	412	455	447
291	CERC2121	*C. eucalypticola*	China, HN	*Eucalyptus* sp.	201	218	255	262	300	325	349	412	455	447
292	CERC2122	*C. eucalypticola*	China, HN	*Eucalyptus* sp.	201	218	255	262	300	325	349	412	455	447
293	CERC2123	*C. eucalypticola*	China, HN	*Eucalyptus* sp.	201	218	255	262	300	325	349	412	455	447
294	CERC2124	*C. eucalypticola*	China, HN	*Eucalyptus* sp.	201	218	255	262	300	325	349	412	455	447
295	CERC2125	*C. eucalypticola*	China, HN	*Eucalyptus* sp.	201	218	255	262	300	325	349	412	455	447
296	CERC2116	*C. eucalypticola*	China, HN	*Eucalyptus* sp.	206	218	252	262	300	325	349	412	455	420
297	CERC2117	*C. eucalypticola*	China, HN	*Eucalyptus* sp.	206	218	252	262	300	325	349	412	455	420
298	CERC2118	*C. eucalypticola*	China, HN	*Eucalyptus* sp.	206	218	252	262	300	325	349	412	455	420
299	CERC2119	*C. eucalypticola*	China, HN	*Eucalyptus* sp.	206	218	252	262	300	325	349	412	455	420
300	CERC2120	*C. eucalypticola*	China, HN	*Eucalyptus* sp.	206	218	252	262	300	325	349	412	455	420
301	CERC2128	*C. eucalypticola*	China, HN	*Eucalyptus* sp.	206	218	252	250	300	325	343	412	455	447
302	CERC5510	*C. eucalypticola*	China, YN	*Eucalyptus* sp.	201	218	249	262	300	325	349	412	455	420
303	CERC5512	*C. eucalypticola*	China, YN	*Eucalyptus* sp.	201	218	249	262	300	325	349	412	455	420
304	CERC5515	*C. eucalypticola*	China, YN	*Eucalyptus* sp.	201	218	249	262	300	325	349	412	455	420
305	CMW4781	*C. eucalypticola*	Congo	*Eucalyptus* clone	201	218	249	250	300	325	346	412	450	426
306	CMW4786	*C. eucalypticola*	Congo	*Eucalyptus* clone	201	218	249	250	300	325	346	412	450	426
307	CMW4787	*C. eucalypticola*	Congo	*Eucalyptus* clone	201	218	249	250	300	325	346	412	450	426
308	CMW4788	*C. eucalypticola*	Congo	*Eucalyptus* clone	201	218	249	250	300	325	346	412	450	426
309	CMW4791	*C. eucalypticola*	Congo	*Eucalyptus* clone	201	218	249	250	300	325	346	412	450	426
310	CMW4797	*C. eucalypticola*	Congo	*Eucalyptus* clone	201	218	249	250	300	325	346	412	450	426
311	CMW4799	*C. eucalypticola*	Congo	*Eucalyptus* clone	201	218	249	250	300	325	346	412	450	426
312	CMW4800	*C. eucalypticola*	Congo	*Eucalyptus* clone	201	218	249	250	300	325	346	412	450	426
313	CMW4803	*C. eucalypticola*	Congo	*Eucalyptus* clone	201	218	249	250	300	325	346	412	450	426
314	CMW4808	*C. eucalypticola*	Congo	*Eucalyptus* clone	201	218	249	250	300	325	346	412	450	426
315	CMW4809	*C. eucalypticola*	Congo	*Eucalyptus* clone	201	218	249	250	300	325	346	412	450	426
316	CMW4748	*C. eucalypticola*	Congo	*Eucalyptus* clone	201	218	237	250	300	325	346	412	447	426

317	CMW4782	*C. eucalypticola*	Congo	*Eucalyptus* clone	201	218	237	250	300	325	346	412	447	426
318	CMW4785	*C. eucalypticola*	Congo	*Eucalyptus* clone	201	218	237	250	300	325	346	412	447	426
319	CMW4794	*C. eucalypticola*	Congo	*Eucalyptus* clone	201	218	237	250	300	325	346	412	447	426
320	CMW4795	*C. eucalypticola*	Congo	*Eucalyptus* clone	201	218	237	250	300	325	346	412	447	426
321	CMW4796	*C. eucalypticola*	Congo	*Eucalyptus* clone	201	218	237	250	300	325	346	412	447	426
322	CMW4802	*C. eucalypticola*	Congo	*Eucalyptus* clone	201	218	237	250	300	325	346	412	447	426
323	CMW4804	*C. eucalypticola*	Congo	*Eucalyptus* clone	201	218	237	250	300	325	346	412	447	426
324	CMW4806	*C. eucalypticola*	Congo	*Eucalyptus* clone	201	218	237	250	300	325	346	412	447	426
325	CMW4807	*C. eucalypticola*	Congo	*Eucalyptus* clone	201	218	237	250	300	325	346	412	447	426
326	CMW4810	*C. eucalypticola*	Congo	*Eucalyptus* clone	201	218	237	250	300	325	346	412	447	426
327	CMW14628	*C. eucalypticola*	Indonesia	*Eucalyptus* sp.	201	218	249	262	300	325	349	412	455	420
328	CMW18563	*C. eucalypticola*	Indonesia	*Eucalyptus* sp.	201	218	249	262	300	325	349	412	455	420
329	CMW18564	*C. eucalypticola*	Indonesia	*Eucalyptus* sp.	201	218	249	262	300	325	349	412	455	420
330	CMW18572	*C. eucalypticola*	Indonesia	*Eucalyptus* sp.	201	218	249	262	300	325	349	412	455	420
331	CMW18573	*C. eucalypticola*	Indonesia	*Eucalyptus* sp.	201	218	249	262	300	325	349	412	455	420
332	CMW20621	*C. eucalypticola*	Indonesia	*Eucalyptus* sp.	201	218	249	262	300	325	349	412	455	420
333	CMW20626	*C. eucalypticola*	Indonesia	*Eucalyptus* sp.	201	218	249	262	300	325	349	412	455	420
334	CMW20628	*C. eucalypticola*	Indonesia	*Eucalyptus* sp.	201	218	249	262	300	325	349	412	455	420
335	CMW20629	*C. eucalypticola*	Indonesia	*Eucalyptus* sp.	201	218	249	262	300	325	349	412	455	420
336	CMW20636	*C. eucalypticola*	Indonesia	*Eucalyptus* sp.	201	218	249	262	300	325	349	412	455	420
337	CMW20637	*C. eucalypticola*	Indonesia	*Eucalyptus* sp.	201	218	249	262	300	325	349	412	455	420
338	CMW20640	*C. eucalypticola*	Indonesia	*Eucalyptus* sp.	201	218	249	262	300	325	349	412	455	420
339	CMW20641	*C. eucalypticola*	Indonesia	*Eucalyptus* sp.	201	218	249	262	300	325	349	412	455	420
340	CMW20663	*C. eucalypticola*	Indonesia	*Eucalyptus* sp.	201	218	249	262	300	325	349	412	455	420
341	CMW20665	*C. eucalypticola*	Indonesia	*Eucalyptus* sp.	201	218	249	262	300	325	349	412	455	420
342	CMW21034	*C. manginecans*	Indonesia	*Eucalyptus* sp.	201	218	249	262	300	325	349	412	455	420
343	CMW20632	*C. eucalypticola*	Indonesia	*Eucalyptus* sp.	201	221	249	262	300	325	349	412	455	420
344	CMW20972	*C. manginecans*	Indonesia	*Eucalyptus* sp.	206	218	243	262	300	325	346	412	447	456
345	CMW20977	*C. manginecans*	Indonesia	*Eucalyptus* sp.	206	218	243	262	300	325	346	412	447	456
346	CMW18577	*C. eucalypticola*	Indonesia	*Eucalyptus* sp.	206	221	249	262	300	325	349	418	447	453
347	CMW20638	*C. eucalypticola*	Indonesia	*Eucalyptus* sp.	206	221	249	262	300	325	349	418	447	450
348	CMW20648	*C. eucalypticola*	Indonesia	*Eucalyptus* sp.	206	221	249	262	294	325	349	418	447	453
349	CMW21044	*C. manginecans*	Indonesia	*Eucalyptus* sp.	206	221	243	262	300	325	349	412	447	453

350	CMW21046	*C. manginecans*	Indonesia	*Eucalyptus* sp.	206	221	243	262	300	325	349	412	447	453
351	CMW21028	*C. manginecans*	Indonesia	*Eucalyptus* sp.	206	221	243	262	300	325	349	418	447	453
352	CMW21048	*C. manginecans*	Indonesia	*Eucalyptus* sp.	206	221	243	262	300	325	349	418	447	453
353	CMW21025	*C. eucalypticola*	Indonesia	*Eucalyptus* sp.	206	221	243	262	303	325	349	418	447	453
354	CMW21049	*C. eucalypticola*	Indonesia	*Eucalyptus* sp.	206	221	243	262	303	325	349	418	447	453
355	CMW13030	*C. eucalypticola*	South Africa	*Eucalyptus* sp.	201	218	249	250	300	325	349	412	452	447
356	CMW15062	*C. eucalypticola*	South Africa	*Eucalyptus* sp.	201	218	249	250	300	325	349	412	452	447
357	CMW10000	*C. eucalypticola*	South Africa	*Eucalyptus grandis*	201	218	252	250	300	325	349	412	450	447
358	CMW12277	*C. eucalypticola*	South Africa	*E. grandis*	201	218	252	250	300	325	349	412	450	447
359	CMW12666	*C. eucalypticola*	South Africa	*E. grandis*	201	218	252	250	300	325	349	412	450	447
360	CMW13019	*C. eucalypticola*	South Africa	*Eucalyptus* sp.	201	218	252	250	300	325	349	412	450	447
361	CMW13022	*C. eucalypticola*	South Africa	*Eucalyptus* sp.	201	218	252	250	300	325	349	412	450	447
362	CMW13023	*C. eucalypticola*	South Africa	*Eucalyptus* sp.	201	218	252	250	300	325	349	412	450	447
363	CMW13027	*C. eucalypticola*	South Africa	*Eucalyptus* sp.	201	218	252	250	300	325	349	412	450	447
364	CMW13028	*C. eucalypticola*	South Africa	*Eucalyptus* sp.	201	218	252	250	300	325	349	412	450	447
365	CMW13040	*C. eucalypticola*	South Africa	*Eucalyptus* sp.	201	218	252	250	300	325	349	412	450	447
366	CMW13041	*C. eucalypticola*	South Africa	*Eucalyptus* sp.	201	218	252	250	300	325	349	412	450	447
367	CMW15061	*C. eucalypticola*	South Africa	*E. grandis*	201	218	252	250	300	325	349	412	450	447
368	CMW9998	*C. eucalypticola*	South Africa	*E. grandis*	201	218	252	250	300	325	349	412	450	447
369	CMW12276	*C. eucalypticola*	South Africa	*E. grandis*	201	218	252	250	300	325	349	412	452	447
370	CMW15054	*C. eucalypticola*	South Africa	*E. grandis*	198	218	249	250	300	325	349	412	450	453
371	CMW11700	*C. eucalypticola*	South Africa	*E. grandis*	198	218	249	250	300	325	349	412	450	447
372	CMW15053	*C. eucalypticola*	South Africa	*E. grandis*	198	218	249	250	300	325	349	412	450	447
373	CMW15056	*C. eucalypticola*	South Africa	*E. grandis*	198	218	249	250	300	325	349	412	452	447
374	CMW15066	*C. eucalypticola*	South Africa	*E. grandis*	198	218	249	250	300	325	349	412	452	447
375	CMW15067	*C. eucalypticola*	South Africa	*E. grandis*	198	218	249	250	300	325	349	412	452	447
376	CMW11701	*C. eucalypticola*	South Africa	*E. grandis*	198	218	249	250	300	325	349	412	452	450
377	CMW15055	*C. eucalypticola*	South Africa	*E. grandis*	198	218	252	250	300	325	349	412	450	447
378	CMW15058	*C. eucalypticola*	South Africa	*E. grandis*	198	218	252	250	300	325	349	412	450	447
379	CMW12664	*C. eucalypticola*	South Africa	*E. grandis*	198	218	252	250	300	325	349	412	450	450
380	CMW12665	*C. eucalypticola*	South Africa	*E. grandis*	198	218	252	250	300	325	349	412	450	450
381	CMW12670	*C. eucalypticola*	South Africa	*E. grandis*	198	218	252	250	300	325	349	412	450	450
382	CMW15070	*C. eucalypticola*	South Africa	*E. grandis*	198	218	252	250	300	325	349	412	452	453

383	CMW39447	*C. eucalypticola*	Uruguay	*E. grandis*	201	218	249	250	300	325	349	412	455	444
384	CMW39484	*C. eucalypticola*	Uruguay	*E. grandis*	201	218	249	250	300	325	349	412	455	444
385	CMW39487	*C. eucalypticola*	Uruguay	*E. grandis*	201	218	249	250	300	325	349	412	455	444
386	CMW39488	*C. eucalypticola*	Uruguay	*E. grandis*	201	218	249	250	300	325	343	412	455	444
387	CMW39442	*C. eucalypticola*	Uruguay	*E. grandis*	201	218	249	250	300	325	352	412	455	447
388	CMW39453	*C. eucalypticola*	Uruguay	*E. grandis*	201	218	249	250	294	325	349	412	455	444
389	CMW39441	*C. eucalypticola*	Uruguay	*E. grandis*	201	218	249	250	294	325	352	412	455	447
390	CMW15313	*C. manginecans*	Oman	*Mangifera indica*	206	221	243	250	294	322	346	418	447	438
391	CMW15314	*C. manginecans*	Oman	*M. indica*	206	221	243	250	294	322	346	418	447	438
392	CMW15315	*C. manginecans*	Oman	*M. indica*	206	221	243	250	294	322	346	418	447	438
393	CMW15316	*C. manginecans*	Oman	*M. indica*	206	221	243	250	294	322	346	418	447	438
394	CMW15317	*C. manginecans*	Oman	*M. indica*	206	221	243	250	294	322	346	418	447	438
395	CMW15353	*C. manginecans*	Oman	*M. indica*	206	221	243	250	294	322	346	418	447	438
396	CMW15366	*C. manginecans*	Oman	*M. indica*	206	221	243	250	294	322	346	418	447	438
397	CMW15369	*C. manginecans*	Oman	*M. indica*	206	221	243	250	294	322	346	418	447	438
398	CMW15371	*C. manginecans*	Oman	*M. indica*	206	221	243	250	294	322	346	418	447	438
399	CMW15377	*C. manginecans*	Oman	*M. indica*	206	221	243	250	294	322	346	418	447	438
400	CMW15381	*C. manginecans*	Oman	*M. indica*	206	221	243	250	294	322	346	418	447	438
401	CMW15382	*C. manginecans*	Oman	*M. indica*	206	221	243	250	294	322	346	418	447	438
402	CMW15384	*C. manginecans*	Oman	*M. indica*	206	221	243	250	294	322	346	418	447	438
403	CMW15385	*C. manginecans*	Oman	*M. indica*	206	221	243	250	294	322	346	418	447	438
404	CMW15391	*C. manginecans*	Oman	*M. indica*	206	221	243	250	294	322	346	418	447	438
405	CMW17567	*C. manginecans*	Pakistan	*M. indica*	206	221	243	250	294	322	346	418	447	438
406	CMW23628	*C. manginecans*	Pakistan	*M. indica*	206	221	243	250	294	322	346	418	447	438
407	CMW23630	*C. manginecans*	Pakistan	*M. indica*	206	221	243	250	294	322	346	418	447	438
408	CMW23637	*C. manginecans*	Pakistan	*M. indica*	206	221	243	250	294	322	346	418	447	438
409	CMW23642	*C. manginecans*	Pakistan	*M. indica*	206	221	243	250	294	322	346	418	447	438
410	CMW23643	*C. manginecans*	Pakistan	*M. indica*	206	221	243	250	294	322	346	418	447	438
411	CERC7783	*C. eucalypticola*	China, SC	*Punica granatum*	201	218	249	262	300	325	349	412	455	420
412	CERC7795	*C. eucalypticola*	China, SC	*P. granatum*	201	218	249	262	300	325	349	412	455	420
413	CERC7806	*C. eucalypticola*	China, SC	*P. granatum*	201	218	249	262	300	325	349	412	455	420
414	CERC7832	*C. eucalypticola*	China, SC	*P. granatum*	201	218	249	262	300	325	349	412	455	420
415	CERC7836	*C. eucalypticola*	China, SC	*P. granatum*	201	218	249	262	300	325	349	412	455	420

416	CERC7845	*C. eucalypticola*	China, SC	*P. granatum*	201	218	249	262	300	325	349	412	455	420
417	CERC7847	*C. eucalypticola*	China, SC	*P. granatum*	201	218	249	262	300	325	349	412	455	420
418	CERC7852	*C. eucalypticola*	China, SC	*P. granatum*	201	218	249	262	300	325	349	412	455	420
419	CERC7853	*C. eucalypticola*	China, SC	*P. granatum*	201	218	249	262	300	325	349	412	455	420
420	CERC7822	*C. eucalypticola*	China, SC	*P. granatum*	201	218	249	262	300	325	349	412	455	447
421	CERC7804	*C. eucalypticola*	China, SC	*P. granatum*	201	218	249	262	300	325	349	412	458	420
422	CERC7810	*C. eucalypticola*	China, SC	*P. granatum*	201	218	249	262	303	325	349	412	455	420
423	CERC7802	*C. eucalypticola*	China, SC	*P. granatum*	201	218	249	250	300	325	349	412	455	420
424	CERC7785	*C. eucalypticola*	China, SC	*P. granatum*	201	218	252	250	300	325	349	412	455	447
425	CERC7789	*C. eucalypticola*	China, SC	*P. granatum*	201	218	252	250	300	325	349	412	455	447
426	CERC7791	*C. eucalypticola*	China, SC	*P. granatum*	201	218	252	250	300	325	349	412	455	447
427	CERC7793	*C. eucalypticola*	China, SC	*P. granatum*	201	218	252	250	300	325	349	412	455	447
428	CERC7798	*C. eucalypticola*	China, SC	*P. granatum*	201	218	252	250	300	325	349	412	455	447
429	CERC7800	*C. eucalypticola*	China, SC	*P. granatum*	201	218	252	250	300	325	349	412	455	447
430	CERC7808	*C. eucalypticola*	China, SC	*P. granatum*	201	218	252	250	300	325	349	412	455	447
431	CERC7812	*C. eucalypticola*	China, SC	*P. granatum*	201	218	252	250	300	325	349	412	455	447
432	CERC7814	*C. eucalypticola*	China, SC	*P. granatum*	201	218	252	250	300	325	349	412	455	447
433	CERC7816	*C. eucalypticola*	China, SC	*P. granatum*	201	218	252	250	300	325	349	412	455	447
434	CERC7818	*C. eucalypticola*	China, SC	*P. granatum*	201	218	252	250	300	325	349	412	455	447
435	CERC7824	*C. eucalypticola*	China, SC	*P. granatum*	201	218	252	250	300	325	349	412	455	447
436	CERC7828	*C. eucalypticola*	China, SC	*P. granatum*	201	218	252	250	300	325	349	412	455	447
437	CERC7830	*C. eucalypticola*	China, SC	*P. granatum*	201	218	252	250	300	325	349	412	455	447
438	CERC7838	*C. eucalypticola*	China, SC	*P. granatum*	201	218	252	250	300	325	349	412	455	447
439	CERC7840	*C. eucalypticola*	China, SC	*P. granatum*	201	218	252	250	300	325	349	412	455	447
440	CERC7841	*C. eucalypticola*	China, SC	*P. granatum*	201	218	252	250	300	325	349	412	455	447
441	CERC7850	*C. eucalypticola*	China, SC	*P. granatum*	201	218	252	250	300	325	349	412	455	447
442	CERC7826	*C. eucalypticola*	China, SC	*P. granatum*	198	218	249	262	300	325	349	412	455	420
443	CERC7843	*C. eucalypticola*	China, SC	*P. granatum*	198	218	249	262	300	325	349	412	455	420
444	CERC3576	*C. eucalypticola*	China, YN	*P. granatum*	201	218	249	262	300	325	349	412	455	420
445	CERC3577	*C. eucalypticola*	China, YN	*P. granatum*	201	218	249	262	300	325	349	412	455	420
446	CERC3597	*C. eucalypticola*	China, YN	*P. granatum*	201	218	249	262	300	325	349	412	455	420
447	CERC5488	*C. eucalypticola*	China, YN	*P. granatum*	201	218	249	262	300	325	349	412	455	420
448	CERC5493	*C. eucalypticola*	China, YN	*P. granatum*	201	218	249	262	300	325	349	412	455	420

449	CERC7741	*C. eucalypticola*	China, YN	*P. granatum*	201	218	249	262	300	325	349	412	455	420
450	CERC3578	*C. eucalypticola*	China, YN	*P. granatum*	198	218	249	262	300	325	349	412	455	420
451	CERC3580	*C. eucalypticola*	China, YN	*P. granatum*	198	218	249	262	300	325	349	412	455	420
452	CERC3581	*C. eucalypticola*	China, YN	*P. granatum*	198	218	249	262	300	325	349	412	455	420
453	CERC3586	*C. eucalypticola*	China, YN	*P. granatum*	198	218	249	262	300	325	349	412	455	420
454	CERC3588	*C. eucalypticola*	China, YN	*P. granatum*	198	218	249	262	300	325	349	412	455	420
455	CERC3590	*C. eucalypticola*	China, YN	*P. granatum*	198	218	249	262	300	325	349	412	455	420
456	CERC3592	*C. eucalypticola*	China, YN	*P. granatum*	198	218	249	262	300	325	349	412	455	420
457	CERC3595	*C. eucalypticola*	China, YN	*P. granatum*	198	218	249	262	300	325	349	412	455	420
458	CERC3598	*C. eucalypticola*	China, YN	*P. granatum*	198	218	249	262	300	325	349	412	455	420
459	CERC7685	*C. eucalypticola*	China, YN	*P. granatum*	198	218	249	262	300	325	349	412	455	420
460	CERC7687	*C. eucalypticola*	China, YN	*P. granatum*	198	218	249	262	300	325	349	412	455	420
461	CERC7689	*C. eucalypticola*	China, YN	*P. granatum*	198	218	249	262	300	325	349	412	455	420
462	CERC7691	*C. eucalypticola*	China, YN	*P. granatum*	198	218	249	262	300	325	349	412	455	420
463	CERC7692	*C. eucalypticola*	China, YN	*P. granatum*	198	218	249	262	300	325	349	412	455	420
464	CERC7694	*C. eucalypticola*	China, YN	*P. granatum*	198	218	249	262	300	325	349	412	455	420
465	CERC7698	*C. eucalypticola*	China, YN	*P. granatum*	198	218	249	262	300	325	349	412	455	420
466	CERC7699	*C. eucalypticola*	China, YN	*P. granatum*	198	218	249	262	300	325	349	412	455	420
467	CERC7701	*C. eucalypticola*	China, YN	*P. granatum*	198	218	249	262	300	325	349	412	455	420
468	CERC7703	*C. eucalypticola*	China, YN	*P. granatum*	198	218	249	262	300	325	349	412	455	420
469	CERC7705	*C. eucalypticola*	China, YN	*P. granatum*	198	218	249	262	300	325	349	412	455	420
470	CERC7707	*C. eucalypticola*	China, YN	*P. granatum*	198	218	249	262	300	325	349	412	455	420
471	CERC7709	*C. eucalypticola*	China, YN	*P. granatum*	198	218	249	262	300	325	349	412	455	420
472	CERC7711	*C. eucalypticola*	China, YN	*P. granatum*	198	218	249	262	300	325	349	412	455	420
473	CERC7713	*C. eucalypticola*	China, YN	*P. granatum*	198	218	249	262	300	325	349	412	455	420
474	CERC7715	*C. eucalypticola*	China, YN	*P. granatum*	198	218	249	262	300	325	349	412	455	420
475	CERC7717	*C. eucalypticola*	China, YN	*P. granatum*	198	218	249	262	300	325	349	412	455	420
476	CERC7719	*C. eucalypticola*	China, YN	*P. granatum*	198	218	249	262	300	325	349	412	455	420
477	CERC7721	*C. eucalypticola*	China, YN	*P. granatum*	198	218	249	262	300	325	349	412	455	420
478	CERC7727	*C. eucalypticola*	China, YN	*P. granatum*	198	218	249	262	300	325	349	412	455	420
479	CERC7729	*C. eucalypticola*	China, YN	*P. granatum*	198	218	249	262	300	325	349	412	455	420
480	CERC7731	*C. eucalypticola*	China, YN	*P. granatum*	198	218	249	262	300	325	349	412	455	420
481	CERC7733	*C. eucalypticola*	China, YN	*P. granatum*	198	218	249	262	300	325	349	412	455	420

482	CERC7735	*C. eucalypticola*	China, YN	*P. granatum*	198	218	249	262	300	325	349	412	455	420
483	CERC7737	*C. eucalypticola*	China, YN	*P. granatum*	198	218	249	262	300	325	349	412	455	420
484	CERC7739	*C. eucalypticola*	China, YN	*P. granatum*	198	218	249	262	300	325	349	412	455	420
485	CERC7743	*C. eucalypticola*	China, YN	*P. granatum*	198	218	249	262	300	325	349	412	455	420
486	CERC7745	*C. eucalypticola*	China, YN	*P. granatum*	198	218	249	262	300	325	349	412	455	420
487	CERC7747	*C. eucalypticola*	China, YN	*P. granatum*	198	218	249	262	300	325	349	412	455	420
488	CERC7749	*C. eucalypticola*	China, YN	*P. granatum*	198	218	249	262	300	325	349	412	455	420
489	CERC7751	*C. eucalypticola*	China, YN	*P. granatum*	198	218	249	262	300	325	349	412	455	420
490	CERC7753	*C. eucalypticola*	China, YN	*P. granatum*	198	218	249	262	300	325	349	412	455	420
491	CERC7696	*C. eucalypticola*	China, YN	*P. granatum*	198	218	249	266	300	325	349	412	455	420

[a] CMW = Culture collection of the Forestry and Agricultural Biotechnology Institute (FABI), University of Pretoria, Pretoria, South Africa; CERC = Culture collection of China Eucalypt Research Centre (CERC), Chinese Academy of Forestry (CAF), ZhanJiang, GuangDong Province, China.

Supplementary Table S2. List of *Ceratocystis* isolates included in the microsatellite study and the alleles scored at each locus based on results obtained by sequencing.

					**Panel AF**									
				**Primer**	**AF2**	**AF3**	**AF4**	**AF5**	**AF6**	**AF7**	**AF8**	**AF9**	**AF11**	**AF12**
				**Dye**	**VIC**	**NED**	**PET**	**FAM**	**VIC**	**NED**	**PET**	**FAM**	**VIC**	**PET**
				**Colour**	**Green**	**Yellow**	**Red**	**Blue**	**Green**	**Yellow**	**Red**	**Blue**	**Green**	**Red**
				**Bin size in Genescan**	**180~230**	**170~270**	**200~280**	**200~300**	**235~320**	**280~370**	**310-380**	**350~460**	**420~490**	**400-480**
				**Repeat type**	**(AGA)n**	**(CTG)n**	**(ACA)n**	**(GTCA)n**	**(GAG)n**	**(AGC)n**	**(GAG)n**	**(GCA)n**	**(ACA)n**	**(ACA)n**
				**Dilution**	**1/200**	**1/200**	**1.2/200**	**2.5/200**	**1/200**	**1.2/200**	**1/200**	**1/200**	**1/200**	**1/200**
				**Annealing temperature**	**55°C**	**55°C**	**55°C**	**54°C**	**54°C**	**55°C**	**54°C**	**54°C**	**55°C**	**57°C**
				**Taq**	**My Taq**	**My Taq**	**My Taq**	**My Taq**	**My Taq**	**My Taq**	**My Taq**	**My Taq**	**My Taq**	**My Taq**
Nr	**Isolate[a]**	**Species**	**Location**	**Host**										
1	CMW22563	*C. manginecans*	Indonesia	*Acacia mangium*	204	218	250	265	301	330	350	416	458	458

2	CMW22561	*C. manginecans*	Indonesia	*A. mangium*	204	218	250	265	301	327	350	416	458	458
3	CMT66	*C. manginecans*	Indonesia	*A. acacinofora*	210	218	250	265	310	330	350	416	449	458
4	CMW22564	*C. manginecans*	Indonesia	*A. mangium*	210	218	244	265	301	330	350	416	449	458
5	CMT25	*C. manginecans*	Indonesia	*A. acacinofora*	210	218	244	265	301	330	350	416	449	464
6	CMT26	*C. manginecans*	Indonesia	*A. acacinofora*	210	218	244	265	301	330	350	416	449	464
7	CMT29	*C. manginecans*	Indonesia	*A. acacinofora*	210	218	244	265	301	330	350	416	449	464
8	CMT1	*C. manginecans*	Indonesia	*A. acacinofora*	210	218	244	265	301	330	350	422	449	458
9	CMT10	*C. manginecans*	Indonesia	*A. acacinofora*	210	218	244	265	301	330	350	422	449	458
10	CMT100	*C. manginecans*	Indonesia	*A. acacinofora*	210	218	244	265	301	330	350	422	449	458
11	CMT101	*C. manginecans*	Indonesia	*A. acacinofora*	210	218	244	265	301	330	350	422	449	458
12	CMT102	*C. manginecans*	Indonesia	*A. acacinofora*	210	218	244	265	301	330	350	422	449	458
13	CMT103	*C. manginecans*	Indonesia	*A. acacinofora*	210	218	244	265	301	330	350	422	449	458
14	CMT11	*C. manginecans*	Indonesia	*A. acacinofora*	210	218	244	265	301	330	350	422	449	458
15	CMT12	*C. manginecans*	Indonesia	*A. acacinofora*	210	218	244	265	301	330	350	422	449	458
16	CMT13	*C. manginecans*	Indonesia	*A. acacinofora*	210	218	244	265	301	330	350	422	449	458
17	CMT15	*C. manginecans*	Indonesia	*A. acacinofora*	210	218	244	265	301	330	350	422	449	458
18	CMT16	*C. manginecans*	Indonesia	*A. acacinofora*	210	218	244	265	301	330	350	422	449	458
19	CMT17	*C. manginecans*	Indonesia	*A. acacinofora*	210	218	244	265	301	330	350	422	449	458
20	CMT18	*C. manginecans*	Indonesia	*A. acacinofora*	210	218	244	265	301	330	350	422	449	458
21	CMT2	*C. manginecans*	Indonesia	*A. acacinofora*	210	218	244	265	301	330	350	422	449	458
22	CMT20	*C. manginecans*	Indonesia	*A. acacinofora*	210	218	244	265	301	330	350	422	449	458
23	CMT21	*C. manginecans*	Indonesia	*A. acacinofora*	210	218	244	265	301	330	350	422	449	458
24	CMT22	*C. manginecans*	Indonesia	*A. acacinofora*	210	218	244	265	301	330	350	422	449	458
25	CMT24	*C. manginecans*	Indonesia	*A. acacinofora*	210	218	244	265	301	330	350	422	449	458
26	CMT28	*C. manginecans*	Indonesia	*A. acacinofora*	210	218	244	265	301	330	350	422	449	458
27	CMT3	*C. manginecans*	Indonesia	*A. acacinofora*	210	218	244	265	301	330	350	422	449	458
28	CMT30	*C. manginecans*	Indonesia	*A. acacinofora*	210	218	244	265	301	330	350	422	449	458
29	CMT31	*C. manginecans*	Indonesia	*A. acacinofora*	210	218	244	265	301	330	350	422	449	458
30	CMT33	*C. manginecans*	Indonesia	*A. acacinofora*	210	218	244	265	301	330	350	422	449	458
31	CMT35	*C. manginecans*	Indonesia	*A. acacinofora*	210	218	244	265	301	330	350	422	449	458
32	CMT36	*C. manginecans*	Indonesia	*A. acacinofora*	210	218	244	265	301	330	350	422	449	458
33	CMT37	*C. manginecans*	Indonesia	*A. acacinofora*	210	218	244	265	301	330	350	422	449	458
34	CMT38	*C. manginecans*	Indonesia	*A. acacinofora*	210	218	244	265	301	330	350	422	449	458
35	CMT39	*C. manginecans*	Indonesia	*A. acacinofora*	210	218	244	265	301	330	350	422	449	458

36	CMT5	*C. manginecans*	Indonesia	*A. acacinofora*	210	218	244	265	301	330	350	422	449	458
37	CMT6	*C. manginecans*	Indonesia	*A. acacinofora*	210	218	244	265	301	330	350	422	449	458
38	CMT7	*C. manginecans*	Indonesia	*A. acacinofora*	210	218	244	265	301	330	350	422	449	458
39	CMT8	*C. manginecans*	Indonesia	*A. acacinofora*	210	218	244	265	301	330	350	422	449	458
40	CMT9	*C. manginecans*	Indonesia	*A. acacinofora*	210	218	244	265	301	330	350	422	449	458
41	CMT94	*C. manginecans*	Indonesia	*A. acacinofora*	210	218	244	265	301	330	350	422	449	458
42	CMT95	*C. manginecans*	Indonesia	*A. acacinofora*	210	218	244	265	301	330	350	422	449	458
43	CMT96	*C. manginecans*	Indonesia	*A. acacinofora*	210	218	244	265	301	330	350	422	449	458
44	CMT98	*C. manginecans*	Indonesia	*A. acacinofora*	210	218	244	265	301	330	350	422	449	458
45	CMT99	*C. manginecans*	Indonesia	*A. acacinofora*	210	218	244	265	301	330	350	422	449	458
46	CMT14	*C. manginecans*	Indonesia	*A. acacinofora*	210	218	244	265	301	330	350	422	449	999
47	CMT4	*C. manginecans*	Indonesia	*A. acacinofora*	210	218	244	265	301	330	350	422	449	999
48	CMW22587	*C. manginecans*	Indonesia	*A. mangium*	210	218	244	265	295	330	350	422	449	458
49	CMW22588	*C. manginecans*	Indonesia	*A. mangium*	210	218	244	265	295	330	350	422	449	458
50	CMW22589	*C. manginecans*	Indonesia	*A. mangium*	210	218	244	265	295	330	350	422	449	458
51	CMW22590	*C. manginecans*	Indonesia	*A. mangium*	210	218	244	265	295	330	350	422	449	458
52	CMW22591	*C. manginecans*	Indonesia	*A. mangium*	210	218	244	265	295	330	350	422	449	458
53	CMW22593	*C. manginecans*	Indonesia	*A. mangium*	210	218	244	265	295	330	350	422	449	458
54	CMW22594	*C. manginecans*	Indonesia	*A. mangium*	210	218	244	265	295	330	350	422	449	458
55	CMW22595	*C. manginecans*	Indonesia	*A. mangium*	210	218	244	265	295	330	350	422	449	458
56	CMW22596	*C. manginecans*	Indonesia	*A. mangium*	210	218	244	265	295	330	350	422	449	458
57	CMW22597	*C. manginecans*	Indonesia	*A. mangium*	210	218	244	265	295	330	350	422	449	458
58	CMW22598	*C. manginecans*	Indonesia	*A. mangium*	210	218	244	265	295	330	350	422	449	458
59	CMW22618	*C. manginecans*	Indonesia	*A. mangium*	210	218	244	265	295	330	350	422	449	461
60	CMW22619	*C. manginecans*	Indonesia	*A. mangium*	210	218	244	265	295	330	350	422	449	461
61	CMW22621	*C. manginecans*	Indonesia	*A. mangium*	210	218	244	265	295	330	350	422	449	461
62	CMW22622	*C. manginecans*	Indonesia	*A. mangium*	210	218	244	265	295	330	350	422	449	461
63	CMW22623	*C. manginecans*	Indonesia	*A. mangium*	210	218	244	265	295	330	350	422	449	461
64	CMW22625	*C. manginecans*	Indonesia	*A. mangium*	210	218	244	265	295	330	350	422	449	461
65	CMW22626	*C. manginecans*	Indonesia	*A. mangium*	210	218	244	265	295	330	350	422	449	461
66	CMT64	*C. manginecans*	Indonesia	*A. acacinofora*	210	218	244	265	295	330	350	422	449	461
67	CMT65	*C. manginecans*	Indonesia	*A. acacinofora*	210	218	244	265	295	330	350	422	449	461
68	CMT67	*C. manginecans*	Indonesia	*A. acacinofora*	210	218	244	265	295	330	350	422	449	461
69	CMT32	*C. manginecans*	Indonesia	*A. acacinofora*	210	218	244	265	295	330	347	422	449	458

70	CMW22581	*C. manginecans*	Indonesia	*A. mangium*	210	221	244	265	295	330	350	422	449	458
71	CMW22560	*C. manginecans*	Indonesia	*A. mangium*	210	221	244	265	295	330	347	422	449	461
72	CMW22565	*C. manginecans*	Indonesia	*A. mangium*	210	221	244	265	295	330	347	422	449	461
73	CMW22566	*C. manginecans*	Indonesia	*A. mangium*	210	221	244	265	295	330	347	422	449	461
74	CMW22567	*C. manginecans*	Indonesia	*A. mangium*	210	221	244	265	295	330	347	422	449	461
75	CMW22568	*C. manginecans*	Indonesia	*A. mangium*	210	221	244	265	295	330	347	422	449	461
76	CMW22569	*C. manginecans*	Indonesia	*A. mangium*	210	221	244	265	295	330	347	422	449	461
77	CMW22570	*C. manginecans*	Indonesia	*A. mangium*	210	221	244	265	295	330	347	422	449	461
78	CMW22571	*C. manginecans*	Indonesia	*A. mangium*	210	221	244	265	295	330	347	422	449	461
79	CMW22572	*C. manginecans*	Indonesia	*A. mangium*	210	221	244	265	295	330	347	422	449	461
80	CMW22573	*C. manginecans*	Indonesia	*A. mangium*	210	221	244	265	295	330	347	422	449	461
81	CMW22574	*C. manginecans*	Indonesia	*A. mangium*	210	221	244	265	295	330	347	422	449	461
82	CMW22575	*C. manginecans*	Indonesia	*A. mangium*	210	221	244	265	295	330	347	422	449	461
83	CMW22576	*C. manginecans*	Indonesia	*A. mangium*	210	221	244	265	295	330	347	422	449	461
84	CMW22577	*C. manginecans*	Indonesia	*A. mangium*	210	221	244	265	295	330	347	422	449	461
85	CMW22578	*C. manginecans*	Indonesia	*A. mangium*	210	221	244	265	295	330	347	422	449	461
86	CMW22579	*C. manginecans*	Indonesia	*A. mangium*	210	221	244	265	295	330	347	422	449	461
87	CMW22580	*C. manginecans*	Indonesia	*A. mangium*	210	221	244	265	295	330	347	422	449	461
88	CMW22582	*C. manginecans*	Indonesia	*A. mangium*	210	221	244	265	295	327	350	422	449	458
89	CMW22583	*C. manginecans*	Indonesia	*A. mangium*	210	221	244	265	295	327	350	422	449	458
90	CMW22584	*C. manginecans*	Indonesia	*A. mangium*	210	221	244	265	295	327	350	422	449	458
91	CMW22585	*C. manginecans*	Indonesia	*A. mangium*	210	221	244	265	295	327	350	422	449	458
92	CMW22600	*C. manginecans*	Indonesia	*A. mangium*	210	221	244	265	295	327	350	422	449	458
93	CMW22601	*C. manginecans*	Indonesia	*A. mangium*	210	221	244	265	295	327	350	422	449	458
94	CMW22603	*C. manginecans*	Indonesia	*A. mangium*	210	221	244	265	295	327	350	422	449	458
95	CMW22606	*C. manginecans*	Indonesia	*A. mangium*	210	221	244	265	295	327	350	422	449	458
96	CMW22607	*C. manginecans*	Indonesia	*A. mangium*	210	221	244	265	295	327	350	422	449	458
97	CMW22608	*C. manginecans*	Indonesia	*A. mangium*	210	221	244	265	295	327	350	422	449	458
98	CMW22609	*C. manginecans*	Indonesia	*A. mangium*	210	221	244	265	295	327	350	422	449	458
99	CMW22612	*C. manginecans*	Indonesia	*A. mangium*	210	221	244	265	295	327	350	422	449	458
100	CMW22613	*C. manginecans*	Indonesia	*A. mangium*	210	221	244	265	295	327	350	422	449	458
101	CMW22614	*C. manginecans*	Indonesia	*A. mangium*	210	221	244	265	295	327	350	422	449	458
102	CMW22615	*C. manginecans*	Indonesia	*A. mangium*	210	221	244	265	295	327	350	422	449	458
103	CMW22616	*C. manginecans*	Indonesia	*A. mangium*	210	221	244	265	295	327	350	422	449	458

104	CMW22617	*C. manginecans*	Indonesia	*A. mangium*	210	221	244	265	295	327	350	422	449	458
105	CMW22599	*C. manginecans*	Indonesia	*A. mangium*	210	221	244	253	295	327	347	422	449	461
106	CMW22602	*C. manginecans*	Indonesia	*A. mangium*	210	221	244	253	295	327	347	422	449	461
107	CMW22604	*C. manginecans*	Indonesia	*A. mangium*	210	221	244	253	295	327	347	422	449	461
108	CMW22605	*C. manginecans*	Indonesia	*A. mangium*	210	221	244	253	295	327	347	422	449	461
109	CMW22610	*C. manginecans*	Indonesia	*A. mangium*	210	221	244	253	295	327	347	422	449	461
110	CMW22611	*C. manginecans*	Indonesia	*A. mangium*	210	221	244	253	295	327	347	422	449	461
111	CMW41202	*C. manginecans*	Malaysia	*A. mangium*	204	221	244	253	295	330	350	422	449	461
112	CMW41190	*C. manginecans*	Malaysia	*A. mangium*	204	221	244	253	295	327	347	422	449	458
113	CMW42003	*C. manginecans*	Malaysia	*A. mangium*	210	218	244	253	295	327	347	422	449	458
114	CMW41203	*C. eucalypticola*	Malaysia	*A. mangium*	210	221	244	253	301	330	350	422	449	458
115	CMW39172	*C. manginecans*	Malaysia	*A. mangium*	210	221	244	253	295	330	347	416	449	458
116	CMW41147	*C. manginecans*	Malaysia	*A. mangium*	210	221	244	253	295	330	347	416	449	446
117	CMW41168	*C. manginecans*	Malaysia	*A. mangium*	210	221	244	253	295	330	347	416	449	446
118	CMW38751	*C. manginecans*	Malaysia	*A. mangium*	210	221	244	253	295	330	347	422	449	458
119	CMW38754	*C. manginecans*	Malaysia	*A. mangium*	210	221	244	253	295	330	347	422	449	458
120	CMW41155	*C. manginecans*	Malaysia	*A. mangium*	210	221	244	253	295	330	347	422	449	458
121	CMW41171	*C. manginecans*	Malaysia	*A. mangium*	210	221	244	253	295	330	347	422	449	458
122	CMW41193	*C. manginecans*	Malaysia	*A. mangium*	210	221	244	253	295	330	347	422	449	458
123	CMW41152	*C. manginecans*	Malaysia	*A. mangium*	210	221	244	253	295	330	347	422	449	446
124	CMW41160	*C. manginecans*	Malaysia	*A. mangium*	210	221	244	253	295	330	347	422	449	446
125	CMW41181	*C. manginecans*	Malaysia	*A. mangium*	210	221	244	253	295	330	347	422	449	446
126	CMW42006	*C. manginecans*	Malaysia	*A. mangium*	210	221	244	253	295	330	347	422	449	461
127	CMW38750	*C. manginecans*	Malaysia	*A. mangium*	210	221	244	253	295	327	347	422	449	458
128	CMW38753	*C. manginecans*	Malaysia	*A. mangium*	210	221	244	253	295	327	347	422	449	458
129	CMW39136	*C. manginecans*	Malaysia	*A. mangium*	210	221	244	253	295	327	347	422	449	458
130	CMW39141	*C. manginecans*	Malaysia	*A. mangium*	210	221	244	253	295	327	347	422	449	458
131	CMW39152	*C. manginecans*	Malaysia	*A. mangium*	210	221	244	253	295	327	347	422	449	458
132	CMW39155	*C. manginecans*	Malaysia	*A. mangium*	210	221	244	253	295	327	347	422	449	458
133	CMW39157	*C. manginecans*	Malaysia	*A. mangium*	210	221	244	253	295	327	347	422	449	458
134	CMW39162	*C. manginecans*	Malaysia	*A. mangium*	210	221	244	253	295	327	347	422	449	458
135	CMW39168	*C. manginecans*	Malaysia	*A. mangium*	210	221	244	253	295	327	347	422	449	458
136	CMW39171	*C. manginecans*	Malaysia	*A. mangium*	210	221	244	253	295	327	347	422	449	458
137	CMW39180	*C. manginecans*	Malaysia	*A. mangium*	210	221	244	253	295	327	347	422	449	458

138	CMW39182	*C. manginecans*	Malaysia	*A. mangium*	210	221	244	253	295	327	347	422	449	458
139	CMW41138	*C. manginecans*	Malaysia	*A. mangium*	210	221	244	253	295	327	347	422	449	458
140	CMW41149	*C. manginecans*	Malaysia	*A. mangium*	210	221	244	253	295	327	347	422	449	458
141	CMW41157	*C. manginecans*	Malaysia	*A. mangium*	210	221	244	253	295	327	347	422	449	458
142	CMW41170	*C. manginecans*	Malaysia	*A. mangium*	210	221	244	253	295	327	347	422	449	458
143	CMW41173	*C. manginecans*	Malaysia	*A. mangium*	210	221	244	253	295	327	347	422	449	458
144	CMW41174	*C. manginecans*	Malaysia	*A. mangium*	210	221	244	253	295	327	347	422	449	458
145	CMW41182	*C. manginecans*	Malaysia	*A. mangium*	210	221	244	253	295	327	347	422	449	458
146	CMW41194	*C. manginecans*	Malaysia	*A. mangium*	210	221	244	253	295	327	347	422	449	458
147	CMW39144	*C. manginecans*	Malaysia	*A. mangium*	210	221	244	253	295	327	347	422	449	446
148	CMW39149	*C. manginecans*	Malaysia	*A. mangium*	210	221	244	253	295	327	347	422	449	446
149	CMW39161	*C. manginecans*	Malaysia	*A. mangium*	210	221	244	253	295	327	347	422	449	446
150	CMW39173	*C. manginecans*	Malaysia	*A. mangium*	210	221	244	253	295	327	347	422	449	446
151	CMW39174	*C. manginecans*	Malaysia	*A. mangium*	210	221	244	253	295	327	347	422	449	446
152	CMW39176	*C. manginecans*	Malaysia	*A. mangium*	210	221	244	253	295	327	347	422	449	446
153	CMW41140	*C. manginecans*	Malaysia	*A. mangium*	210	221	244	253	295	327	347	422	449	446
154	CMW41143	*C. manginecans*	Malaysia	*A. mangium*	210	221	244	253	295	327	347	422	449	446
155	CMW41150	*C. manginecans*	Malaysia	*A. mangium*	210	221	244	253	295	327	347	422	449	446
156	CMW41164	*C. manginecans*	Malaysia	*A. mangium*	210	221	244	253	295	327	347	422	449	446
157	CMW41169	*C. manginecans*	Malaysia	*A. mangium*	210	221	244	253	295	327	347	422	449	446
158	CMW39139	*C. manginecans*	Malaysia	*A. mangium*	210	221	244	253	295	327	347	422	449	461
159	CMW41139	*C. manginecans*	Malaysia	*A. mangium*	210	221	244	253	295	327	347	422	449	464
160	CMW41148	*C. manginecans*	Malaysia	*A. mangium*	210	221	244	253	295	327	347	422	446	446
161	CMW41146	*C. manginecans*	Malaysia	*A. mangium*	210	221	244	253	295	324	347	422	449	458
162	CMW36164	*C. manginecans*	Malaysia	*A. mangium*	210	221	244	253	292	330	347	422	449	458
163	CMW39153	*C. manginecans*	Malaysia	*A. mangium*	210	221	244	253	292	330	347	422	449	458
164	CMW41142	*C. manginecans*	Malaysia	*A. mangium*	213	221	244	253	295	327	347	422	449	458
165	CMW41159	*C. manginecans*	Malaysia	*A. mangium*	213	221	244	253	295	327	347	422	449	458
166	CMW49238	*C. eucalypticola*	Brazil	*Eucalyptus* sp.	204	218	250	253	301	330	350	416	458	428
167	CMW49250	*C. eucalypticola*	Brazil	*Eucalyptus* sp.	204	218	250	253	301	330	350	419	458	449
168	CMW49237	*C. eucalypticola*	Brazil	*Eucalyptus* sp.	204	218	250	253	301	330	353	419	452	428
169	CMW49233	*C. eucalypticola*	Brazil	*Eucalyptus* sp.	204	218	250	253	301	330	353	419	452	428
170	CMW49252	*C. eucalypticola*	Brazil	*Eucalyptus* sp.	201	218	250	253	301	330	350	416	455	434
171	CMW49253	*C. eucalypticola*	Brazil	*Eucalyptus* sp.	201	218	250	253	301	330	350	416	455	434

172	CMW49239	*C. eucalypticola*	Brazil	*Eucalyptus* sp.	201	218	250	253	301	330	350	416	455	437
173	CMW49236	*C. eucalypticola*	Brazil	*Eucalyptus* sp.	201	218	250	253	301	330	350	419	455	428
174	CMW49254	*C. eucalypticola*	Brazil	*Eucalyptus* sp.	201	218	250	253	301	330	350	419	455	449
175	CMW49255	*C. eucalypticola*	Brazil	*Eucalyptus* sp.	201	218	250	253	301	330	350	419	455	449
176	CMW4903	*C. eucalypticola*	Brazil	*Eucalyptus* sp.	201	218	250	253	301	330	350	419	455	452
177	CMW49251	*C. eucalypticola*	Brazil	*Eucalyptus* sp.	201	218	250	253	301	330	350	419	455	452
178	CMW49235	*C. eucalypticola*	Brazil	*Eucalyptus* sp.	201	218	250	253	295	330	350	416	455	434
179	CERC2453	*C. eucalypticola*	China, FJ	*Eucalyptus* sp.	204	218	250	265	301	330	350	416	458	452
180	CERC2454	*C. eucalypticola*	China, FJ	*Eucalyptus* sp.	204	218	250	265	301	330	350	416	458	452
181	CERC2455	*C. eucalypticola*	China, FJ	*Eucalyptus* sp.	204	218	250	265	301	330	350	416	458	452
182	CERC2476	*C. eucalypticola*	China, FJ	*Eucalyptus* sp.	204	218	250	265	301	330	350	416	458	452
183	CERC2477	*C. eucalypticola*	China, FJ	*Eucalyptus* sp.	204	218	250	265	301	330	350	416	458	452
184	CERC2478	*C. eucalypticola*	China, FJ	*Eucalyptus* sp.	204	218	250	265	301	330	350	416	458	452
185	CERC2479	*C. eucalypticola*	China, FJ	*Eucalyptus* sp.	204	218	250	265	301	330	350	416	458	452
186	CERC2480	*C. eucalypticola*	China, FJ	*Eucalyptus* sp.	201	218	250	265	301	330	350	416	458	452
187	CERC2487	*C. eucalypticola*	China, FJ	*Eucalyptus* sp.	201	218	250	253	301	330	350	416	458	455
188	CERC2488	*C. eucalypticola*	China, FJ	*Eucalyptus* sp.	201	218	250	253	301	330	350	416	458	455
189	CERC2489	*C. eucalypticola*	China, FJ	*Eucalyptus* sp.	201	218	250	253	301	330	350	416	458	455
190	CERC2490	*C. eucalypticola*	China, FJ	*Eucalyptus* sp.	201	218	250	253	301	330	350	416	458	455
191	CERC2491	*C. eucalypticola*	China, FJ	*Eucalyptus* sp.	201	218	250	253	301	330	350	416	458	455
192	CERC2545	*C. eucalypticola*	China, GD	*Eucalyptus* sp.	204	218	250	265	301	330	344	416	458	425
193	CERC2546	*C. eucalypticola*	China, GD	*Eucalyptus* sp.	204	218	250	265	301	330	344	416	458	425
194	CERC2612	*C. eucalypticola*	China, GD	*Eucalyptus* sp.	204	218	250	265	301	330	344	416	452	452
195	CMW24667	*C. eucalypticola*	China, GD	*Eucalyptus* sp.	204	218	250	253	301	330	350	416	458	452
196	CERC2555	*C. eucalypticola*	China, GD	*Eucalyptus* sp.	204	218	250	253	301	330	350	416	452	452
197	CERC2556	*C. eucalypticola*	China, GD	*Eucalyptus* sp.	204	218	250	253	301	330	350	416	452	452
198	CERC2557	*C. eucalypticola*	China, GD	*Eucalyptus* sp.	204	218	250	253	301	330	350	416	452	452
199	CMW24664	*C. eucalypticola*	China, GD	*Eucalyptus* sp.	204	218	253	265	301	330	350	416	458	425
200	CMW24673	*C. eucalypticola*	China, GD	*Eucalyptus* sp.	204	218	253	265	301	330	350	416	458	425
201	CERC2620	*C. eucalypticola*	China, GD	*Eucalyptus* sp.	204	218	253	253	301	330	350	416	452	452
202	CERC2621	*C. eucalypticola*	China, GD	*Eucalyptus* sp.	204	218	253	253	301	330	350	416	452	452
203	CERC2629	*C. manginecans*	China, GD	*Eucalyptus* sp.	204	221	253	253	295	330	347	422	452	458
204	CERC2630	*C. manginecans*	China, GD	*Eucalyptus* sp.	204	221	253	253	295	330	347	422	452	458
205	CERC2631	*C. manginecans*	China, GD	*Eucalyptus* sp.	204	221	253	253	295	330	347	422	452	458

206	CERC2632	*C. manginecans*	China, GD	*Eucalyptus* sp.	204	221	253	253	295	330	347	422	452	458
207	CERC2633	*C. manginecans*	China, GD	*Eucalyptus* sp.	204	221	253	253	295	330	347	422	452	458
208	CERC2634	*C. manginecans*	China, GD	*Eucalyptus* sp.	204	221	253	253	295	330	347	422	452	458
209	CERC2635	*C. manginecans*	China, GD	*Eucalyptus* sp.	204	221	253	253	295	330	347	422	452	458
210	CERC2636	*C. manginecans*	China, GD	*Eucalyptus* sp.	204	221	253	253	295	330	347	422	452	458
211	CERC2637	*C. manginecans*	China, GD	*Eucalyptus* sp.	204	221	253	253	295	330	347	422	452	458
212	CERC2638	*C. manginecans*	China, GD	*Eucalyptus* sp.	204	221	253	253	295	330	347	422	452	458
213	CERC2639	*C. manginecans*	China, GD	*Eucalyptus* sp.	204	221	253	253	295	330	347	422	452	458
214	CERC2640	*C. manginecans*	China, GD	*Eucalyptus* sp.	204	221	253	253	295	330	347	422	452	458
215	CERC2641	*C. manginecans*	China, GD	*Eucalyptus* sp.	204	221	253	253	295	330	347	422	452	458
216	CERC2642	*C. manginecans*	China, GD	*Eucalyptus* sp.	204	221	253	253	295	330	347	422	452	458
217	CERC2643	*C. manginecans*	China, GD	*Eucalyptus* sp.	204	221	253	253	295	330	347	422	452	458
218	CERC2644	*C. manginecans*	China, GD	*Eucalyptus* sp.	204	221	253	253	295	330	347	422	452	458
219	CERC2645	*C. manginecans*	China, GD	*Eucalyptus* sp.	204	221	253	253	295	330	347	422	452	458
220	CERC2624	*C. manginecans*	China, GD	*Eucalyptus* sp.	210	221	250	253	301	330	347	422	449	458
221	CERC2625	*C. manginecans*	China, GD	*Eucalyptus* sp.	210	221	250	253	301	330	347	422	449	458
222	CERC2626	*C. manginecans*	China, GD	*Eucalyptus* sp.	210	221	250	253	301	330	347	422	449	458
223	CERC2589	*C. eucalypticola*	China, GD	*Eucalyptus* sp.	201	218	250	253	301	330	353	416	452	452
224	CERC2590	*C. eucalypticola*	China, GD	*Eucalyptus* sp.	201	218	250	253	301	330	353	416	452	452
225	CERC2591	*C. eucalypticola*	China, GD	*Eucalyptus* sp.	201	218	250	253	301	330	353	416	452	452
226	CERC2592	*C. eucalypticola*	China, GD	*Eucalyptus* sp.	201	218	250	253	301	330	353	416	452	452
227	CERC2613	*C. eucalypticola*	China, GD	*Eucalyptus* sp.	201	218	250	253	301	330	353	416	452	452
228	CERC2614	*C. eucalypticola*	China, GD	*Eucalyptus* sp.	201	218	250	253	301	330	353	416	452	452
229	CERC2615	*C. eucalypticola*	China, GD	*Eucalyptus* sp.	201	218	250	253	301	330	353	416	452	452
230	CERC2616	*C. eucalypticola*	China, GD	*Eucalyptus* sp.	201	218	250	253	301	330	353	416	452	452
231	CERC2617	*C. eucalypticola*	China, GD	*Eucalyptus* sp.	201	218	250	253	301	330	353	416	452	452
232	CERC2618	*C. eucalypticola*	China, GD	*Eucalyptus* sp.	201	218	250	253	301	330	353	416	452	452
233	CERC2619	*C. eucalypticola*	China, GD	*Eucalyptus* sp.	201	218	250	253	301	330	353	416	452	452
234	CERC2622	*C. eucalypticola*	China, GD	*Eucalyptus* sp.	201	218	250	253	301	330	353	416	452	452
235	CERC2623	*C. eucalypticola*	China, GD	*Eucalyptus* sp.	201	218	250	253	301	330	353	416	452	452
236	CERC2627	*C. eucalypticola*	China, GD	*Eucalyptus* sp.	201	218	253	265	301	330	350	416	458	452
237	CERC2628	*C. eucalypticola*	China, GD	*Eucalyptus* sp.	201	218	253	265	301	330	350	416	458	452
238	CERC2593	*C. eucalypticola*	China, GD	*Eucalyptus* sp.	201	218	253	265	301	330	344	416	458	452
239	CERC2594	*C. eucalypticola*	China, GD	*Eucalyptus* sp.	201	218	253	265	301	330	344	416	458	452

240	CERC2595	*C. eucalypticola*	China, GD	*Eucalyptus* sp.	201	218	253	265	301	330	344	416	458	452
241	CERC2596	*C. eucalypticola*	China, GD	*Eucalyptus* sp.	201	218	253	265	301	330	344	416	458	452
242	CERC2597	*C. eucalypticola*	China, GD	*Eucalyptus* sp.	201	218	253	265	301	330	344	416	458	452
243	CERC2598	*C. eucalypticola*	China, GD	*Eucalyptus* sp.	201	218	253	265	301	330	344	416	458	452
244	CERC2599	*C. eucalypticola*	China, GD	*Eucalyptus* sp.	201	218	253	265	301	330	344	416	458	452
245	CERC2587	*C. eucalypticola*	China, GD	*Eucalyptus* sp.	201	218	253	265	301	333	350	416	452	452
246	CERC2588	*C. eucalypticola*	China, GD	*Eucalyptus* sp.	201	218	253	265	301	333	350	416	452	452
247	CERC2606	*C. eucalypticola*	China, GD	*Eucalyptus* sp.	201	218	253	265	301	333	350	416	452	452
248	CERC2607	*C. eucalypticola*	China, GD	*Eucalyptus* sp.	201	218	253	265	301	333	350	416	452	452
249	CERC2608	*C. eucalypticola*	China, GD	*Eucalyptus* sp.	201	218	253	265	301	333	350	416	452	452
250	CERC2009	*C. eucalypticola*	China, GX	*Eucalyptus* sp.	204	218	253	253	301	330	350	416	458	455
251	CERC2010	*C. eucalypticola*	China, GX	*Eucalyptus* sp.	204	218	253	253	301	330	350	416	458	455
252	CERC2011	*C. eucalypticola*	China, GX	*Eucalyptus* sp.	204	218	253	253	301	330	350	416	458	455
253	CERC2012	*C. eucalypticola*	China, GX	*Eucalyptus* sp.	204	218	253	253	301	330	350	416	458	455
254	CERC2013	*C. eucalypticola*	China, GX	*Eucalyptus* sp.	204	218	253	253	301	330	350	416	458	455
255	CERC2016	*C. eucalypticola*	China, GX	*Eucalyptus* sp.	204	218	253	253	301	330	350	416	458	455
256	CERC2017	*C. eucalypticola*	China, GX	*Eucalyptus* sp.	204	218	253	253	301	330	350	416	458	455
257	CERC2103	*C. eucalypticola*	China, HN	*Eucalyptus* sp.	204	218	250	253	301	330	350	416	458	452
258	CERC2104	*C. eucalypticola*	China, HN	*Eucalyptus* sp.	204	218	250	253	301	330	350	416	458	452
259	CERC2105	*C. eucalypticola*	China, HN	*Eucalyptus* sp.	204	218	250	253	301	330	350	416	458	452
260	CERC2106	*C. eucalypticola*	China, HN	*Eucalyptus* sp.	204	218	250	253	301	330	350	416	458	452
261	CERC2107	*C. eucalypticola*	China, HN	*Eucalyptus* sp.	204	218	250	253	301	330	350	416	458	452
262	CERC2109	*C. eucalypticola*	China, HN	*Eucalyptus* sp.	204	218	250	253	301	330	350	416	458	452
263	CERC2110	*C. eucalypticola*	China, HN	*Eucalyptus* sp.	204	218	250	253	301	330	350	416	458	452
264	CERC2111	*C. eucalypticola*	China, HN	*Eucalyptus* sp.	204	218	250	253	301	330	350	416	458	452
265	CERC2113	*C. eucalypticola*	China, HN	*Eucalyptus* sp.	204	218	250	253	301	330	350	416	458	452
266	CERC2115	*C. eucalypticola*	China, HN	*Eucalyptus* sp.	204	218	250	253	301	330	350	416	458	452
267	CERC2068	*C. eucalypticola*	China, HN	*Eucalyptus* sp.	204	218	250	253	301	330	350	416	452	452
268	CERC2070	*C. eucalypticola*	China, HN	*Eucalyptus* sp.	204	218	250	253	301	330	350	416	452	452
269	CERC2074	*C. eucalypticola*	China, HN	*Eucalyptus* sp.	204	218	250	253	301	330	350	416	452	452
270	CERC2076	*C. eucalypticola*	China, HN	*Eucalyptus* sp.	204	218	250	253	301	330	350	416	452	452
271	CERC2079	*C. eucalypticola*	China, HN	*Eucalyptus* sp.	204	218	250	253	301	330	350	416	452	452
272	CERC2080	*C. eucalypticola*	China, HN	*Eucalyptus* sp.	204	218	250	253	301	330	350	416	452	452
273	CERC2082	*C. eucalypticola*	China, HN	*Eucalyptus* sp.	204	218	250	253	301	330	350	416	452	452

274	CERC2084	*C. eucalypticola*	China, HN	*Eucalyptus* sp.	204	218	250	253	301	330	350	416	452	452
275	CERC2085	*C. eucalypticola*	China, HN	*Eucalyptus* sp.	204	218	250	253	301	330	350	416	452	452
276	CERC2088	*C. eucalypticola*	China, HN	*Eucalyptus* sp.	204	218	250	253	301	330	350	416	452	452
277	CERC2089	*C. eucalypticola*	China, HN	*Eucalyptus* sp.	204	218	250	253	301	330	350	416	452	452
278	CERC2097	*C. eucalypticola*	China, HN	*Eucalyptus* sp.	204	218	250	253	301	330	350	416	452	452
279	CERC2101	*C. eucalypticola*	China, HN	*Eucalyptus* sp.	204	218	250	253	301	330	350	416	452	452
280	CERC2102	*C. eucalypticola*	China, HN	*Eucalyptus* sp.	204	218	250	253	301	330	350	416	452	452
281	CERC2190	*C. eucalypticola*	China, HN	*Eucalyptus* sp.	204	218	253	265	301	330	350	416	458	452
282	CERC2191	*C. eucalypticola*	China, HN	*Eucalyptus* sp.	204	218	253	265	301	330	350	416	458	452
283	CERC2192	*C. eucalypticola*	China, HN	*Eucalyptus* sp.	204	218	253	265	301	330	350	416	458	452
284	CERC2193	*C. eucalypticola*	China, HN	*Eucalyptus* sp.	204	218	253	265	301	330	350	416	458	452
285	CERC2194	*C. eucalypticola*	China, HN	*Eucalyptus* sp.	204	218	253	265	301	330	350	416	458	452
286	CERC2195	*C. eucalypticola*	China, HN	*Eucalyptus* sp.	204	218	253	265	301	330	350	416	458	452
287	CERC2196	*C. eucalypticola*	China, HN	*Eucalyptus* sp.	204	218	253	265	301	330	350	416	458	452
288	CERC2126	*C. eucalypticola*	China, HN	*Eucalyptus* sp.	204	218	253	265	301	330	344	416	458	452
289	CERC2127	*C. eucalypticola*	China, HN	*Eucalyptus* sp.	204	218	253	265	301	330	344	416	458	452
290	CERC2129	*C. eucalypticola*	China, HN	*Eucalyptus* sp.	204	218	253	265	301	330	344	416	458	452
291	CERC2121	*C. eucalypticola*	China, HN	*Eucalyptus* sp.	204	218	256	265	301	330	350	416	458	452
292	CERC2122	*C. eucalypticola*	China, HN	*Eucalyptus* sp.	204	218	256	265	301	330	350	416	458	452
293	CERC2123	*C. eucalypticola*	China, HN	*Eucalyptus* sp.	204	218	256	265	301	330	350	416	458	452
294	CERC2124	*C. eucalypticola*	China, HN	*Eucalyptus* sp.	204	218	256	265	301	330	350	416	458	452
295	CERC2125	*C. eucalypticola*	China, HN	*Eucalyptus* sp.	204	218	256	265	301	330	350	416	458	452
296	CERC2116	*C. eucalypticola*	China, HN	*Eucalyptus* sp.	210	218	253	265	301	330	350	416	458	425
297	CERC2117	*C. eucalypticola*	China, HN	*Eucalyptus* sp.	210	218	253	265	301	330	350	416	458	425
298	CERC2118	*C. eucalypticola*	China, HN	*Eucalyptus* sp.	210	218	253	265	301	330	350	416	458	425
299	CERC2119	*C. eucalypticola*	China, HN	*Eucalyptus* sp.	210	218	253	265	301	330	350	416	458	425
300	CERC2120	*C. eucalypticola*	China, HN	*Eucalyptus* sp.	210	218	253	265	301	330	350	416	458	425
301	CERC2128	*C. eucalypticola*	China, HN	*Eucalyptus* sp.	210	218	253	253	301	330	344	416	458	452
302	CERC5510	*C. eucalypticola*	China, YN	*Eucalyptus* sp.	204	218	250	265	301	330	350	416	458	425
303	CERC5512	*C. eucalypticola*	China, YN	*Eucalyptus* sp.	204	218	250	265	301	330	350	416	458	425
304	CERC5515	*C. eucalypticola*	China, YN	*Eucalyptus* sp.	204	218	250	265	301	330	350	416	458	425
305	CMW4781	*C. eucalypticola*	Congo	*Eucalyptus* clone	204	218	250	253	301	330	347	416	452	431
306	CMW4786	*C. eucalypticola*	Congo	*Eucalyptus* clone	204	218	250	253	301	330	347	416	452	431
307	CMW4787	*C. eucalypticola*	Congo	*Eucalyptus* clone	204	218	250	253	301	330	347	416	452	431

308	CMW4788	*C. eucalypticola*	Congo	*Eucalyptus* clone	204	218	250	253	301	330	347	416	452	431
309	CMW4791	*C. eucalypticola*	Congo	*Eucalyptus* clone	204	218	250	253	301	330	347	416	452	431
310	CMW4797	*C. eucalypticola*	Congo	*Eucalyptus* clone	204	218	250	253	301	330	347	416	452	431
311	CMW4799	*C. eucalypticola*	Congo	*Eucalyptus* clone	204	218	250	253	301	330	347	416	452	431
312	CMW4800	*C. eucalypticola*	Congo	*Eucalyptus* clone	204	218	250	253	301	330	347	416	452	431
313	CMW4803	*C. eucalypticola*	Congo	*Eucalyptus* clone	204	218	250	253	301	330	347	416	452	431
314	CMW4808	*C. eucalypticola*	Congo	*Eucalyptus* clone	204	218	250	253	301	330	347	416	452	431
315	CMW4809	*C. eucalypticola*	Congo	*Eucalyptus* clone	204	218	250	253	301	330	347	416	452	431
316	CMW4748	*C. eucalypticola*	Congo	*Eucalyptus* clone	204	218	238	253	301	330	347	416	449	431
317	CMW4782	*C. eucalypticola*	Congo	*Eucalyptus* clone	204	218	238	253	301	330	347	416	449	431
318	CMW4785	*C. eucalypticola*	Congo	*Eucalyptus* clone	204	218	238	253	301	330	347	416	449	431
319	CMW4794	*C. eucalypticola*	Congo	*Eucalyptus* clone	204	218	238	253	301	330	347	416	449	431
320	CMW4795	*C. eucalypticola*	Congo	*Eucalyptus* clone	204	218	238	253	301	330	347	416	449	431
321	CMW4796	*C. eucalypticola*	Congo	*Eucalyptus* clone	204	218	238	253	301	330	347	416	449	431
322	CMW4802	*C. eucalypticola*	Congo	*Eucalyptus* clone	204	218	238	253	301	330	347	416	449	431
323	CMW4804	*C. eucalypticola*	Congo	*Eucalyptus* clone	204	218	238	253	301	330	347	416	449	431
324	CMW4806	*C. eucalypticola*	Congo	*Eucalyptus* clone	204	218	238	253	301	330	347	416	449	431
325	CMW4807	*C. eucalypticola*	Congo	*Eucalyptus* clone	204	218	238	253	301	330	347	416	449	431
326	CMW4810	*C. eucalypticola*	Congo	*Eucalyptus* clone	204	218	238	253	301	330	347	416	449	431
327	CMW14628	*C. eucalypticola*	Indonesia	*Eucalyptus* sp.	204	218	250	265	301	330	350	416	458	425
328	CMW18563	*C. eucalypticola*	Indonesia	*Eucalyptus* sp.	204	218	250	265	301	330	350	416	458	425
329	CMW18564	*C. eucalypticola*	Indonesia	*Eucalyptus* sp.	204	218	250	265	301	330	350	416	458	425
330	CMW18572	*C. eucalypticola*	Indonesia	*Eucalyptus* sp.	204	218	250	265	301	330	350	416	458	425
331	CMW18573	*C. eucalypticola*	Indonesia	*Eucalyptus* sp.	204	218	250	265	301	330	350	416	458	425
332	CMW20621	*C. eucalypticola*	Indonesia	*Eucalyptus* sp.	204	218	250	265	301	330	350	416	458	425
333	CMW20626	*C. eucalypticola*	Indonesia	*Eucalyptus* sp.	204	218	250	265	301	330	350	416	458	425
334	CMW20628	*C. eucalypticola*	Indonesia	*Eucalyptus* sp.	204	218	250	265	301	330	350	416	458	425
335	CMW20629	*C. eucalypticola*	Indonesia	*Eucalyptus* sp.	204	218	250	265	301	330	350	416	458	425
336	CMW20636	*C. eucalypticola*	Indonesia	*Eucalyptus* sp.	204	218	250	265	301	330	350	416	458	425
337	CMW20637	*C. eucalypticola*	Indonesia	*Eucalyptus* sp.	204	218	250	265	301	330	350	416	458	425
338	CMW20640	*C. eucalypticola*	Indonesia	*Eucalyptus* sp.	204	218	250	265	301	330	350	416	458	425
339	CMW20641	*C. eucalypticola*	Indonesia	*Eucalyptus* sp.	204	218	250	265	301	330	350	416	458	425
340	CMW20663	*C. eucalypticola*	Indonesia	*Eucalyptus* sp.	204	218	250	265	301	330	350	416	458	425
341	CMW20665	*C. eucalypticola*	Indonesia	*Eucalyptus* sp.	204	218	250	265	301	330	350	416	458	425

342	CMW21034	*C. manginecans*	Indonesia	*Eucalyptus* sp.	204	218	250	265	301	330	350	416	458	425
343	CMW20632	*C. eucalypticola*	Indonesia	*Eucalyptus* sp.	204	221	250	265	301	330	350	416	458	425
344	CMW20972	*C. manginecans*	Indonesia	*Eucalyptus* sp.	210	218	244	265	301	330	347	416	449	461
345	CMW20977	*C. manginecans*	Indonesia	*Eucalyptus* sp.	210	218	244	265	301	330	347	416	449	461
346	CMW18577	*C. eucalypticola*	Indonesia	*Eucalyptus* sp.	210	221	250	265	301	330	350	422	449	458
347	CMW20638	*C. eucalypticola*	Indonesia	*Eucalyptus* sp.	210	221	250	265	301	330	350	422	449	455
348	CMW20648	*C. eucalypticola*	Indonesia	*Eucalyptus* sp.	210	221	250	265	295	330	350	422	449	458
349	CMW21044	*C. manginecans*	Indonesia	*Eucalyptus* sp.	210	221	244	265	301	330	350	416	449	458
350	CMW21046	*C. manginecans*	Indonesia	*Eucalyptus* sp.	210	221	244	265	301	330	350	416	449	458
351	CMW21028	*C. manginecans*	Indonesia	*Eucalyptus* sp.	210	221	244	265	301	330	350	422	449	458
352	CMW21048	*C. manginecans*	Indonesia	*Eucalyptus* sp.	210	221	244	265	301	330	350	422	449	458
353	CMW21025	*C. eucalypticola*	Indonesia	*Eucalyptus* sp.	210	221	244	265	304	330	350	422	449	458
354	CMW21049	*C. eucalypticola*	Indonesia	*Eucalyptus* sp.	210	221	244	265	304	330	350	422	449	458
355	CMW13030	*C. eucalypticola*	South Africa	*Eucalyptus* sp.	204	218	250	253	301	330	350	416	455	452
356	CMW15062	*C. eucalypticola*	South Africa	*Eucalyptus grandis*	204	218	250	253	301	330	350	416	455	452
357	CMW10000	*C. eucalypticola*	South Africa	*E. grandis*	204	218	253	253	301	330	350	416	452	452
358	CMW12277	*C. eucalypticola*	South Africa	*E. grandis*	204	218	253	253	301	330	350	416	452	452
359	CMW12666	*C. eucalypticola*	South Africa	*E. grandis*	204	218	253	253	301	330	350	416	452	452
360	CMW13019	*C. eucalypticola*	South Africa	*Eucalyptus* sp.	204	218	253	253	301	330	350	416	452	452
361	CMW13022	*C. eucalypticola*	South Africa	*Eucalyptus* sp.	204	218	253	253	301	330	350	416	452	452
362	CMW13023	*C. eucalypticola*	South Africa	*Eucalyptus* sp.	204	218	253	253	301	330	350	416	452	452
363	CMW13027	*C. eucalypticola*	South Africa	*Eucalyptus* sp.	204	218	253	253	301	330	350	416	452	452
364	CMW13028	*C. eucalypticola*	South Africa	*Eucalyptus* sp.	204	218	253	253	301	330	350	416	452	452
365	CMW13040	*C. eucalypticola*	South Africa	*Eucalyptus* sp.	204	218	253	253	301	330	350	416	452	452
366	CMW13041	*C. eucalypticola*	South Africa	*Eucalyptus* sp.	204	218	253	253	301	330	350	416	452	452
367	CMW15061	*C. eucalypticola*	South Africa	*E. grandis*	204	218	253	253	301	330	350	416	452	452
368	CMW9998	*C. eucalypticola*	South Africa	*E. grandis*	204	218	253	253	301	330	350	416	452	452
369	CMW12276	*C. eucalypticola*	South Africa	*E. grandis*	204	218	253	253	301	330	350	416	455	452
370	CMW15054	*C. eucalypticola*	South Africa	*E. grandis*	201	218	250	253	301	330	350	416	452	458
371	CMW11700	*C. eucalypticola*	South Africa	*E. grandis*	201	218	250	253	301	330	350	416	452	452
372	CMW15053	*C. eucalypticola*	South Africa	*E. grandis*	201	218	250	253	301	330	350	416	452	452
373	CMW15056	*C. eucalypticola*	South Africa	*E. grandis*	201	218	250	253	301	330	350	416	455	452
374	CMW15066	*C. eucalypticola*	South Africa	*E. grandis*	201	218	250	253	301	330	350	416	455	452
375	CMW15067	*C. eucalypticola*	South Africa	*E. grandis*	201	218	250	253	301	330	350	416	455	452

376	CMW11701	*C. eucalypticola*	South Africa	*E. grandis*	201	218	250	253	301	330	350	416	455	455
377	CMW15055	*C. eucalypticola*	South Africa	*E. grandis*	201	218	253	253	301	330	350	416	452	452
378	CMW15058	*C. eucalypticola*	South Africa	*E. grandis*	201	218	253	253	301	330	350	416	452	452
379	CMW12664	*C. eucalypticola*	South Africa	*E. grandis*	201	218	253	253	301	330	350	416	452	455
380	CMW12665	*C. eucalypticola*	South Africa	*E. grandis*	201	218	253	253	301	330	350	416	452	455
381	CMW12670	*C. eucalypticola*	South Africa	*E. grandis*	201	218	253	253	301	330	350	416	452	455
382	CMW15070	*C. eucalypticola*	South Africa	*E. grandis*	201	218	253	253	301	330	350	416	455	458
383	CMW39447	*C. eucalypticola*	Uruguay	*E. grandis*	204	218	250	253	301	330	350	416	458	449
384	CMW39484	*C. eucalypticola*	Uruguay	*E. grandis*	204	218	250	253	301	330	350	416	458	449
385	CMW39487	*C. eucalypticola*	Uruguay	*E. grandis*	204	218	250	253	301	330	350	416	458	449
386	CMW39488	*C. eucalypticola*	Uruguay	*E. grandis*	204	218	250	253	301	330	344	416	458	449
387	CMW39442	*C. eucalypticola*	Uruguay	*E. grandis*	204	218	250	253	301	330	353	416	458	452
388	CMW39453	*C. eucalypticola*	Uruguay	*E. grandis*	204	218	250	253	295	330	350	416	458	449
389	CMW39441	*C. eucalypticola*	Uruguay	*E. grandis*	204	218	250	253	295	330	353	416	458	452
390	CMW15313	*C. manginecans*	Oman	*Mangifera indica*	210	221	244	253	295	327	347	422	449	438
391	CMW15314	*C. manginecans*	Oman	*M. indica*	210	221	244	253	295	327	347	422	449	438
392	CMW15315	*C. manginecans*	Oman	*M. indica*	210	221	244	253	295	327	347	422	449	438
393	CMW15316	*C. manginecans*	Oman	*M. indica*	210	221	244	253	295	327	347	422	449	438
394	CMW15317	*C. manginecans*	Oman	*M. indica*	210	221	244	253	295	327	347	422	449	438
395	CMW15353	*C. manginecans*	Oman	*M. indica*	210	221	244	253	295	327	347	422	449	438
396	CMW15366	*C. manginecans*	Oman	*M. indica*	210	221	244	253	295	327	347	422	449	438
397	CMW15369	*C. manginecans*	Oman	*M. indica*	210	221	244	253	295	327	347	422	449	438
398	CMW15371	*C. manginecans*	Oman	*M. indica*	210	221	244	253	295	327	347	422	449	438
399	CMW15377	*C. manginecans*	Oman	*M. indica*	210	221	244	253	295	327	347	422	449	438
400	CMW15381	*C. manginecans*	Oman	*M. indica*	210	221	244	253	295	327	347	422	449	438
401	CMW15382	*C. manginecans*	Oman	*M. indica*	210	221	244	253	295	327	347	422	449	438
402	CMW15384	*C. manginecans*	Oman	*M. indica*	210	221	244	253	295	327	347	422	449	438
403	CMW15385	*C. manginecans*	Oman	*M. indica*	210	221	244	253	295	327	347	422	449	438
404	CMW15391	*C. manginecans*	Oman	*M. indica*	210	221	244	253	295	327	347	422	449	438
405	CMW17567	*C. manginecans*	Pakistan	*M. indica*	210	221	244	253	295	327	347	422	449	438
406	CMW23628	*C. manginecans*	Pakistan	*M. indica*	210	221	244	253	295	327	347	422	449	438
407	CMW23630	*C. manginecans*	Pakistan	*M. indica*	210	221	244	253	295	327	347	422	449	438
408	CMW23637	*C. manginecans*	Pakistan	*M. indica*	210	221	244	253	295	327	347	422	449	438
409	CMW23642	*C. manginecans*	Pakistan	*M. indica*	210	221	244	253	295	327	347	422	449	438

410	CMW23643	*C. manginecans*	Pakistan	*M. indica*	210	221	244	253	295	327	347	422	449	438
411	CERC7783	*C. eucalypticola*	China, SC	*Punica granatum*	204	218	250	265	301	330	350	416	458	425
412	CERC7795	*C. eucalypticola*	China, SC	*P. granatum*	204	218	250	265	301	330	350	416	458	425
413	CERC7806	*C. eucalypticola*	China, SC	*P. granatum*	204	218	250	265	301	330	350	416	458	425
414	CERC7832	*C. eucalypticola*	China, SC	*P. granatum*	204	218	250	265	301	330	350	416	458	425
415	CERC7836	*C. eucalypticola*	China, SC	*P. granatum*	204	218	250	265	301	330	350	416	458	425
416	CERC7845	*C. eucalypticola*	China, SC	*P. granatum*	204	218	250	265	301	330	350	416	458	425
417	CERC7847	*C. eucalypticola*	China, SC	*P. granatum*	204	218	250	265	301	330	350	416	458	425
418	CERC7852	*C. eucalypticola*	China, SC	*P. granatum*	204	218	250	265	301	330	350	416	458	425
419	CERC7853	*C. eucalypticola*	China, SC	*P. granatum*	204	218	250	265	301	330	350	416	458	425
420	CERC7822	*C. eucalypticola*	China, SC	*P. granatum*	204	218	250	265	301	330	350	416	458	452
421	CERC7804	*C. eucalypticola*	China, SC	*P. granatum*	204	218	250	265	301	330	350	416	461	425
422	CERC7810	*C. eucalypticola*	China, SC	*P. granatum*	204	218	250	265	304	330	350	416	458	425
423	CERC7802	*C. eucalypticola*	China, SC	*P. granatum*	204	218	250	253	301	330	350	416	458	425
424	CERC7785	*C. eucalypticola*	China, SC	*P. granatum*	204	218	253	253	301	330	350	416	458	452
425	CERC7789	*C. eucalypticola*	China, SC	*P. granatum*	204	218	253	253	301	330	350	416	458	452
426	CERC7791	*C. eucalypticola*	China, SC	*P. granatum*	204	218	253	253	301	330	350	416	458	452
427	CERC7793	*C. eucalypticola*	China, SC	*P. granatum*	204	218	253	253	301	330	350	416	458	452
428	CERC7798	*C. eucalypticola*	China, SC	*P. granatum*	204	218	253	253	301	330	350	416	458	452
429	CERC7800	*C. eucalypticola*	China, SC	*P. granatum*	204	218	253	253	301	330	350	416	458	452
430	CERC7808	*C. eucalypticola*	China, SC	*P. granatum*	204	218	253	253	301	330	350	416	458	452
431	CERC7812	*C. eucalypticola*	China, SC	*P. granatum*	204	218	253	253	301	330	350	416	458	452
432	CERC7814	*C. eucalypticola*	China, SC	*P. granatum*	204	218	253	253	301	330	350	416	458	452
433	CERC7816	*C. eucalypticola*	China, SC	*P. granatum*	204	218	253	253	301	330	350	416	458	452
434	CERC7818	*C. eucalypticola*	China, SC	*P. granatum*	204	218	253	253	301	330	350	416	458	452
435	CERC7824	*C. eucalypticola*	China, SC	*P. granatum*	204	218	253	253	301	330	350	416	458	452
436	CERC7828	*C. eucalypticola*	China, SC	*P. granatum*	204	218	253	253	301	330	350	416	458	452
437	CERC7830	*C. eucalypticola*	China, SC	*P. granatum*	204	218	253	253	301	330	350	416	458	452
438	CERC7838	*C. eucalypticola*	China, SC	*P. granatum*	204	218	253	253	301	330	350	416	458	452
439	CERC7840	*C. eucalypticola*	China, SC	*P. granatum*	204	218	253	253	301	330	350	416	458	452
440	CERC7841	*C. eucalypticola*	China, SC	*P. granatum*	204	218	253	253	301	330	350	416	458	452
441	CERC7850	*C. eucalypticola*	China, SC	*P. granatum*	204	218	253	253	301	330	350	416	458	452
442	CERC7826	*C. eucalypticola*	China, SC	*P. granatum*	201	218	250	265	301	330	350	416	458	425
443	CERC7843	*C. eucalypticola*	China, SC	*P. granatum*	201	218	250	265	301	330	350	416	458	425

444	CERC3576	*C. eucalypticola*	China, YN	*P. granatum*	204	218	250	265	301	330	350	416	458	425
445	CERC3577	*C. eucalypticola*	China, YN	*P. granatum*	204	218	250	265	301	330	350	416	458	425
446	CERC3597	*C. eucalypticola*	China, YN	*P. granatum*	204	218	250	265	301	330	350	416	458	425
447	CERC5488	*C. eucalypticola*	China, YN	*P. granatum*	204	218	250	265	301	330	350	416	458	425
448	CERC5493	*C. eucalypticola*	China, YN	*P. granatum*	204	218	250	265	301	330	350	416	458	425
449	CERC7741	*C. eucalypticola*	China, YN	*P. granatum*	204	218	250	265	301	330	350	416	458	425
450	CERC3578	*C. eucalypticola*	China, YN	*P. granatum*	201	218	250	265	301	330	350	416	458	425
451	CERC3580	*C. eucalypticola*	China, YN	*P. granatum*	201	218	250	265	301	330	350	416	458	425
452	CERC3581	*C. eucalypticola*	China, YN	*P. granatum*	201	218	250	265	301	330	350	416	458	425
453	CERC3586	*C. eucalypticola*	China, YN	*P. granatum*	201	218	250	265	301	330	350	416	458	425
454	CERC3588	*C. eucalypticola*	China, YN	*P. granatum*	201	218	250	265	301	330	350	416	458	425
455	CERC3590	*C. eucalypticola*	China, YN	*P. granatum*	201	218	250	265	301	330	350	416	458	425
456	CERC3592	*C. eucalypticola*	China, YN	*P. granatum*	201	218	250	265	301	330	350	416	458	425
457	CERC3595	*C. eucalypticola*	China, YN	*P. granatum*	201	218	250	265	301	330	350	416	458	425
458	CERC3598	*C. eucalypticola*	China, YN	*P. granatum*	201	218	250	265	301	330	350	416	458	425
459	CERC7685	*C. eucalypticola*	China, YN	*P. granatum*	201	218	250	265	301	330	350	416	458	425
460	CERC7687	*C. eucalypticola*	China, YN	*P. granatum*	201	218	250	265	301	330	350	416	458	425
461	CERC7689	*C. eucalypticola*	China, YN	*P. granatum*	201	218	250	265	301	330	350	416	458	425
462	CERC7691	*C. eucalypticola*	China, YN	*P. granatum*	201	218	250	265	301	330	350	416	458	425
463	CERC7692	*C. eucalypticola*	China, YN	*P. granatum*	201	218	250	265	301	330	350	416	458	425
464	CERC7694	*C. eucalypticola*	China, YN	*P. granatum*	201	218	250	265	301	330	350	416	458	425
465	CERC7698	*C. eucalypticola*	China, YN	*P. granatum*	201	218	250	265	301	330	350	416	458	425
466	CERC7699	*C. eucalypticola*	China, YN	*P. granatum*	201	218	250	265	301	330	350	416	458	425
467	CERC7701	*C. eucalypticola*	China, YN	*P. granatum*	201	218	250	265	301	330	350	416	458	425
468	CERC7703	*C. eucalypticola*	China, YN	*P. granatum*	201	218	250	265	301	330	350	416	458	425
469	CERC7705	*C. eucalypticola*	China, YN	*P. granatum*	201	218	250	265	301	330	350	416	458	425
470	CERC7707	*C. eucalypticola*	China, YN	*P. granatum*	201	218	250	265	301	330	350	416	458	425
471	CERC7709	*C. eucalypticola*	China, YN	*P. granatum*	201	218	250	265	301	330	350	416	458	425
472	CERC7711	*C. eucalypticola*	China, YN	*P. granatum*	201	218	250	265	301	330	350	416	458	425
473	CERC7713	*C. eucalypticola*	China, YN	*P. granatum*	201	218	250	265	301	330	350	416	458	425
474	CERC7715	*C. eucalypticola*	China, YN	*P. granatum*	201	218	250	265	301	330	350	416	458	425
475	CERC7717	*C. eucalypticola*	China, YN	*P. granatum*	201	218	250	265	301	330	350	416	458	425
476	CERC7719	*C. eucalypticola*	China, YN	*P. granatum*	201	218	250	265	301	330	350	416	458	425
477	CERC7721	*C. eucalypticola*	China, YN	*P. granatum*	201	218	250	265	301	330	350	416	458	425

478	CERC7727	*C. eucalypticola*	China, YN	*P. granatum*	201	218	250	265	301	330	350	416	458	425
479	CERC7729	*C. eucalypticola*	China, YN	*P. granatum*	201	218	250	265	301	330	350	416	458	425
480	CERC7731	*C. eucalypticola*	China, YN	*P. granatum*	201	218	250	265	301	330	350	416	458	425
481	CERC7733	*C. eucalypticola*	China, YN	*P. granatum*	201	218	250	265	301	330	350	416	458	425
482	CERC7735	*C. eucalypticola*	China, YN	*P. granatum*	201	218	250	265	301	330	350	416	458	425
483	CERC7737	*C. eucalypticola*	China, YN	*P. granatum*	201	218	250	265	301	330	350	416	458	425
484	CERC7739	*C. eucalypticola*	China, YN	*P. granatum*	201	218	250	265	301	330	350	416	458	425
485	CERC7743	*C. eucalypticola*	China, YN	*P. granatum*	201	218	250	265	301	330	350	416	458	425
486	CERC7745	*C. eucalypticola*	China, YN	*P. granatum*	201	218	250	265	301	330	350	416	458	425
487	CERC7747	*C. eucalypticola*	China, YN	*P. granatum*	201	218	250	265	301	330	350	416	458	425
488	CERC7749	*C. eucalypticola*	China, YN	*P. granatum*	201	218	250	265	301	330	350	416	458	425
489	CERC7751	*C. eucalypticola*	China, YN	*P. granatum*	201	218	250	265	301	330	350	416	458	425
490	CERC7753	*C. eucalypticola*	China, YN	*P. granatum*	201	218	250	265	301	330	350	416	458	425
491	CERC7696	*C. eucalypticola*	China, YN	*P. granatum*	201	218	250	269	301	330	350	416	458	425

[a] CMW = Culture collection of the Forestry and Agricultural Biotechnology Institute (FABI), University of Pretoria, Pretoria, South Africa; CERC = Culture collection of China Eucalypt Research Centre (CERC), Chinese Academy of Forestry (CAF), ZhanJiang, GuangDong Province, China.

Supplementary Table S3. GenBank numbers alleles sequenced in this study.

Number	Microsatellite Marker	Repeats	Sequence length	Genescan length	GenBank number
1	AF2	(AGA)8	201	198	MK029868; MK029869
2		(AGA)9	204	201	MK029870; MK029871
3		(AGA)11	210	206	MK029873; MK029874
4		(AGA)12	213	209	MK029875
5	AF3	(CTG)12	218	218	MK029877; MK029878
6		(CTG)13	221	221	MK029879; MK029880
7	AF4	(CAG)2(ACA)6	238	237	MK029881; MK029882
8		(CAG)2(ACA)8	244	243	MK029883; MK029884
9		(CAG)2(ACA)10	250	249	MK029885; MK029886
10		(CAG)3(ACA)10	253	252	MK029887; MK029888
11		(CAG)3(ACA)11	256	255	MK029889; MK029890
12	AF5	(GTCA)6	253	250	MK029891; MK029892
13		(GTCA)9	265	262	MK029893; MK029894
14		(GTCA)10	269	266	MK029895
15	AF6	(GAG)8	292	291	MK029898; MK029899
16		(GAG)9	295	294	MK029900; MK029901
17		(GAG)11	301	300	MK029903; MK029904
18		(GAG)12	304	303	MK029905; MK029906
19	AF7	(AGC)8	324	319	MK029907
20		(AGC)9	327	322	MK029908; MK029909
21		(AGC)10	330	325	MK029910; MK029911
22		(AGC)11	333	328	MK029912; MK029913
23	AF8	(GAG)9	344	343	MK029914; MK029915
24		(GAG)10	347	346	MK029916; MK029917
25		(GAG)11	350	349	MK029918; MK029919
26		(GAG)12	353	352	MK029920
27	AF9	(GCA)9	416	412	MK029921; MK029922
28		(GCA)10	419	415	MK029923; MK029924
29		(GCA)11	422	418	MK029925; MK029926
30	AF11	(ACA)6	446	444	MK029929
31		(ACA)7	449	447	MK029930; MK029931
32		(ACA)8	452	450	MK029932; MK029935
33		(ACA)9	455	452	MK029936; MK029937
34		(ACA)10	458	455	MK029938; MK029939
35		(ACA)11	461	458	MK029940
36	AF12	(CCACAA)4(CAG)2(CAA)5	425	420	MK029942; MK029943
37		(CCACAA)3(CAG)2(CAA)8	428	423	MK029944; MK029945
38		(CCACAA)3(CAG)2(CAA)9	431	426	MK029946; MK029947
39		(CCACAA)3(CAG)2(CAA)10	434	429	MK029948; MK029949
40		(CCACAA)3(CAG)2(CAA)11	437	432	MK029950

41		(CCACAA)3(CAG)3(CAA)13	446	441	MK029952
42		(CCACAA)3(CAG)2(CAA)15	449	444	MK029954; MK029955
43		(CCACAA)3(CAG)2(CAA)16	452	447	MK029956; MK029957
44		(CCACAA)3(CAG)2(CAA)17	455	450	MK029958; MK029959
45		(CCACAA)3(CAG)3(CAA)17	458	453	MK029960; MK029961
46		(CCACAA)3(CAG)3(CAA)18	461	456	MK029962; MK029963
47		(CCACAA)3(CAG)3(CAA)19	464	459	MK029964
